# CD24 flags anastasis in melanoma cells

**DOI:** 10.1007/s10495-024-01990-1

**Published:** 2024-08-13

**Authors:** Martina H. Vasileva, Anette Bennemann, Karolin Zachmann, Michael P. Schön, Jorge Frank, Vijay Kumar Ulaganathan

**Affiliations:** 1 Department of Dermatology, Venereology and Allergology, University Medical Center Göttingen, Göttingen, Germany; 2 Lower Saxony Institute of Occupational Dermatology, University Medical Center Göttingen, Göttingen, Germany; 3 University of Lorraine, NGERE Unit, Faculté de Médecine, 9 Avenue de La Forêt de Haye, 54505 Vandoeuvre-Lès-Nancy, France; 4 Institut für Medizinische Genetik und Angewandte Genomik, Universitätsklinikum Tübingen, 72076 Tübingen, Germany

**Keywords:** B16-F10, YUMM5.2, Cell Membrane, Apoptosis, Anastasis marker, CD24, Cancer-shed particulates, Melanoma, Metastasis

## Abstract

Anastasis is a phenomenon observed in cancer cells, where cells that have initiated apoptosis are able to recover and survive. This molecular event is increasingly recognized as a potential contributor to cancer metastasis, facilitating the survival and migration of tumor cells. Nevertheless, the identification of a specific surface marker for detecting cancer cells in anastasis remained elusive. Here we report our observation that the cell surface expression of CD24 is preferentially enriched in a non-adherent FSC^low^SSC^high^ melanoma subpopulation, which is generally considered a non-viable population in cultivated melanoma cell lines. More than 90% of non-adherent FSC^low^SSC^high^CD24^+ve^ metastatic melanoma cells exhibited bonafide features of apoptosis on the cell surface and in the nucleus, marking apoptotic or seemingly apoptotic subpopulations of the in vitro cultivated metastatic melanoma cell lines. Unexpectedly, however, the CD24^+ve^ subpopulation, despite being apoptotic, showed evidence of metabolic activity and exhibited proliferative capacities, including anchorage-independent growth, when inoculated in soft agarose growth medium. These findings indicate that apoptotic FSC^low^SSC^high^CD24^+ve^ melanoma subpopulations are capable of reversing the progression of apoptosis. We report CD24 as the first novel cell surface marker for anastasis in melanoma cells.

**Supplementary Information:**

The online version contains supplementary material available at 10.1007/s10495-024-01990-1.

## Introduction

Metastatic melanoma is a highly aggressive and lethal form of skin cancer that can spread to distant organs such as the lungs, liver, and brain [1, 2]. Despite advances in early detection and therapy, melanoma remains a significant health concern. This is primarily due to its tendency to metastasize and develop resistance to therapy. Anchorage-independent growth is one of the hallmarks of aggressive forms of cancer, enabling cancer cells to detach from the primary tumor, invade surrounding tissues, and colonize distant organs [3]. Cancer cell detachment is considered an early step in the process of metastasis [4]. We observed that almost all cancer cell types, including malignant melanoma cells, actively release a large number of detached cells or cellular debris components into the culture supernatants, which we collectively term cancer-shed particulates [5]. These cell membrane-bound particulates are capable of driving DNA-directed protein synthesis in vitro, suggesting that they retain biological activities. In recent years, it has been shown that seemingly apoptotic cells can recover and resume proliferation under certain conditions [6, 7], a process known as anastasis. Furthermore, erroneous or failed apoptosis can even promote melanoma aggressiveness in vivo [8]. However, no cell surface marker has yet been identified to conveniently identify cancer cell subpopulation likely to recover from the brink of apoptotic cell death [9, 10], which promote melanoma aggressiveness.

CD24 is a GPI-anchored membrane protein that is considered a stem cell marker in many human malignancies [11–16] and has recently emerged as a target for cancer immunotherapy [17–21]. Based on our unexpected observation of the cell surface expression of CD24, we here investigated whether CD24 expression is associated with the reversal of apoptosis using B16-F10 and YUMM 5.2 cells as melanoma cellular models.

## Results

### Expression of CD24 is restricted to a subpopulation of melanoma cells

CD24 is expressed in skin tumors, with a strong indication of higher expression in tumor tissues as compared to their tissue of origin (Supplementary Fig. 1). Survival analysis of melanoma patients, stratified by CD24 mRNA expression levels (top 5 percentile), shows a trend towards unfavorable outcome in patients with very high CD24 expression (log rank p-value 0.02) (Supplementary Fig. 2).

**Fig. 1:**
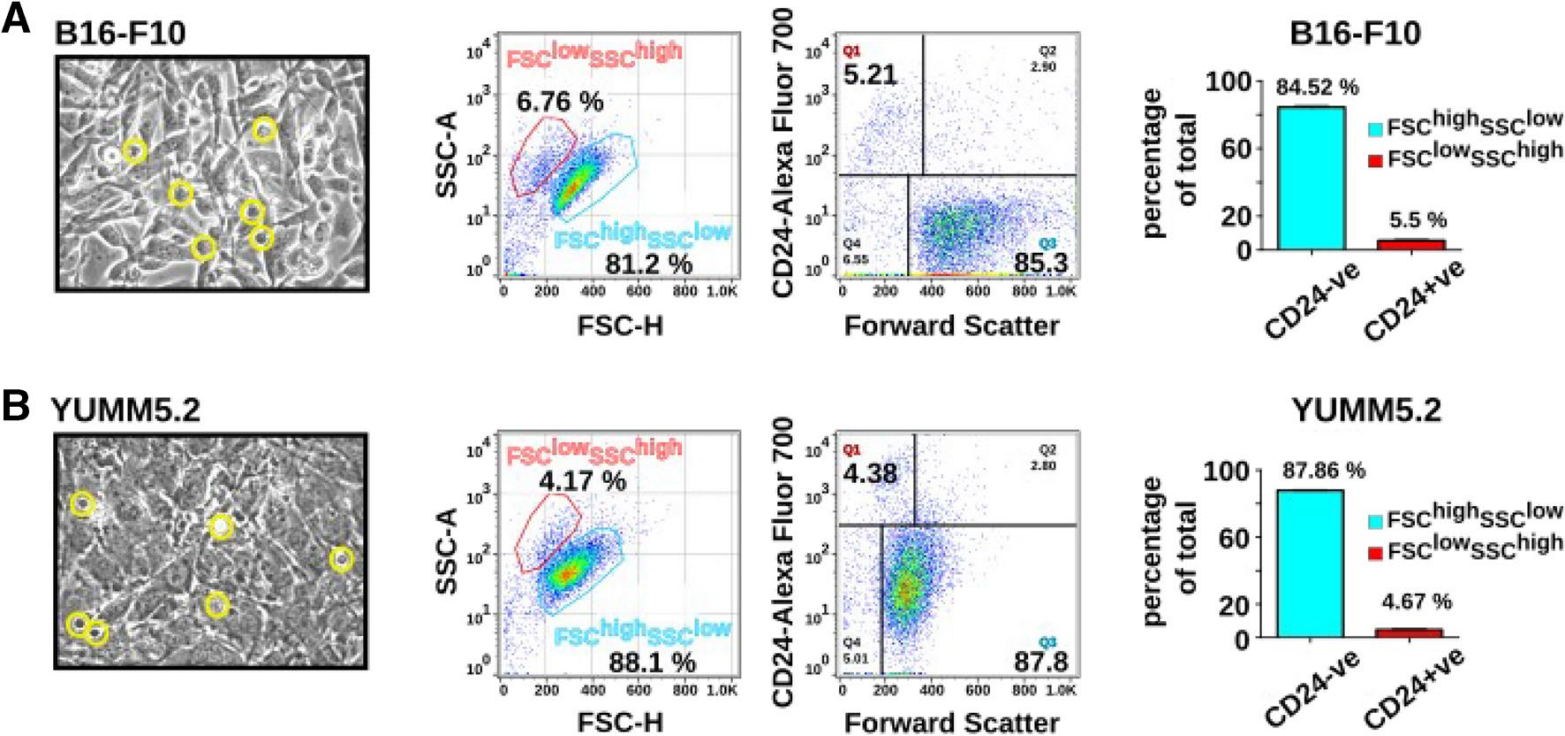
**Surface expression analysis of CD24 in melanoma cell lines. **Transmitted light microscopy images depict B16-F10 (panel A) and YUMM5.2 (panel B) melanoma cell line cultures, with non-adherent and smaller sized subpopulations, indicated by yellow circles (40x magnification). Flow cytometry dot plots show distinct populations in the forward and sideward scatter plots, and the cell surface expression of CD24 in the FSC^low^ subpopulation. The bar histogram quantifies CD24-expressing subpopulations (Results representative of 5 independent experiments)

**Fig. 2:**
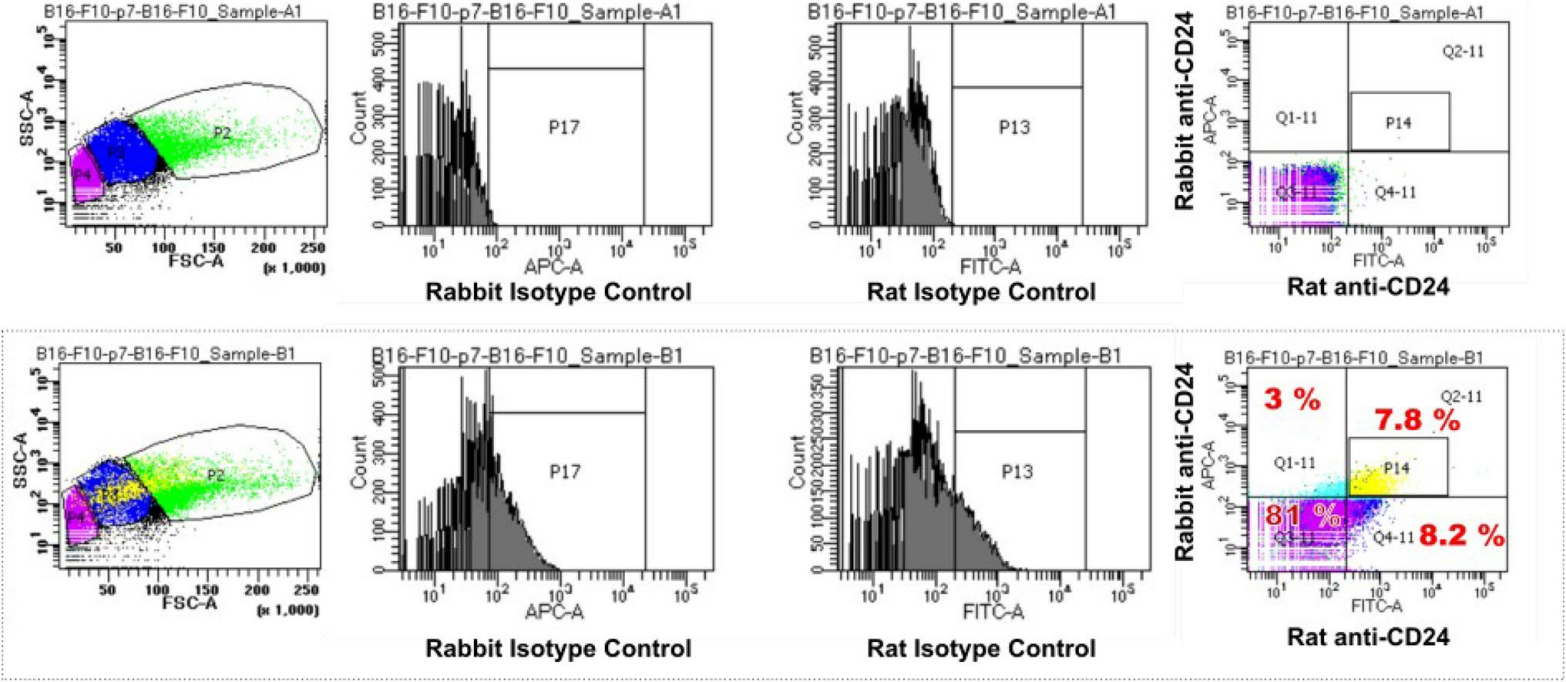
**Combined surface and intracellular expression analysis of CD24.** Flow cytometry dot plots showing the results of combined surface and intracellular staining of CD24 using two different antibodies specific for murine CD24.

**Fig. 3:**
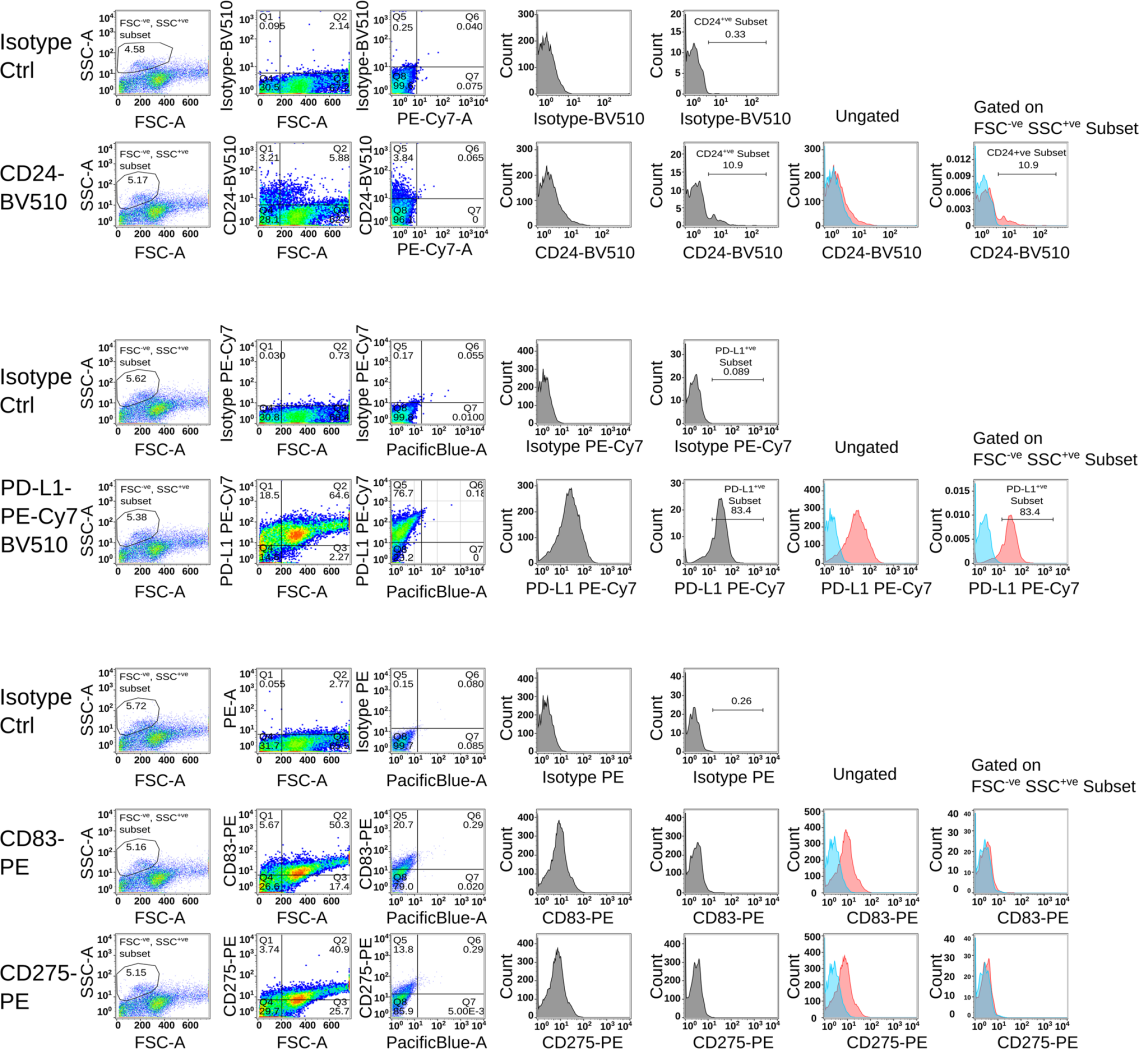
**Comparison of surface expression of CD24, PD-L1 (CD274), CD83 and ICOSLG (CD275) in metastatic melanoma cell line B16-F10. **Flow cytometry dot plots show the surface expression of CD24, PD-L1 (CD274), CD83 and ICOSLG (CD275) on gated and ungated populations

Cell surface expression analysis using two different monoclonal antibodies specific for CD24 (clones M1/69 and 30-F1) revealed a unexpected expression pattern for CD24, restricted to the FSC^low^ and SSC^high^ subpopulations of B16-F10 cells (Supplementary Fig. 3). Interestingly, only about 4–6% of melanoma cells such as B16-F10 and YUMM5.2, showed positivity for CD24, which was found to be exclusively restricted to the FSC^low^ and SSC^high^ subpopulations (Fig. 1). By using rat and rabbit monoclonal antibodies specific for CD24, we were able to detect both surface and intracellular expression of CD24 through combined surface and intracellular staining in B16-F10 (Fig. 2). Approximately 3% of cells displayed exclusive intracellular expression of CD24, while around 8% showed only surface expression. Additionally, approximately 7% of cells exhibited both surface and cytoplasmic staining for CD24. We observed that the phenomenon of distinct expression patterns in the FSC^low^SSC^high^ subpopulation extends also to other transmembrane proteins known to interact with inhibitory receptors of the immune system, such as PD-L1 (CD274), CD83, and ICOSLG (CD275). While CD83 and CD275 showed no detectable expression in the FSC^low^SSC^high^ subpopulation, CD274 expression was also detected in the FSC^low^SSC^high^ subpopulation (Fig. 3).

**Fig. 4:**
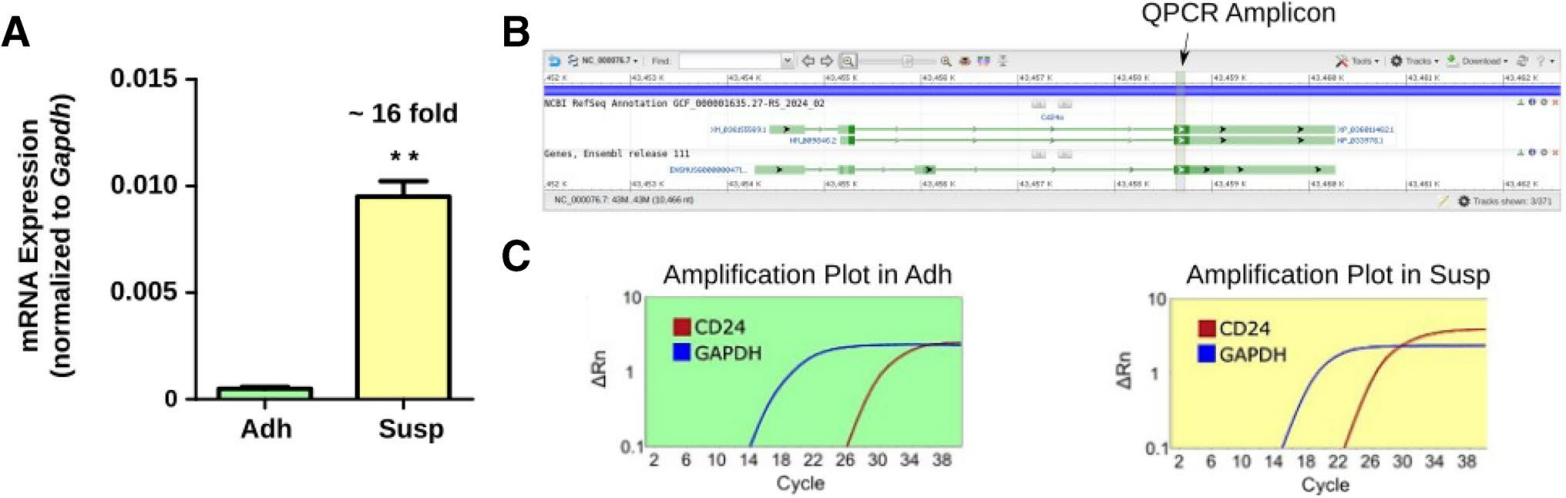
**Expression of mRNA in Susp and Adh subpopulations.** (**A**) Levels of *Cd24* transcript expression in Adh and Susp subpopulations of B16-F10 cells. The fold change is indicated. Expression values are determined using the 2^(-ΔCt) method normalized with respect to *Gapdh* as the housekeeping gene. Paired one-tailed Student’s t-test, *p* < 0.005, n=3. (**B**) Graphical view of the murine CD24 gene, with transcript isoforms shown in green. The qPCR amplicon region, matching the coding sequence common to all three mouse *Cd24 *transcripts, is indicated. (**C**) Amplification plots of the real-time PCR-based quantification of *Cd24* mRNA in Adh (green) and Susp (yellow) cells are shown

Melanoma cell cultures such as B16-F10 and YUMM5.2 that are in the exponential growth phase have some smaller, detached subpopulations on top of the bigger, adherent cells. When collected and analyzed by flow cytometry, more than 70% of all suspension cells from both B16-F10 and YUMM5.2 cell cultures were FSC^low^SSC^high^, hereafter referred to as Susp cells. Of these, about 70% expressed CD24 on the cell surface (Supplementary Fig. 4). In contrast, a large majority of the adherent cell population were mostly FSC^high^SSC^low^, hereafter referred to as Adh cells. Furthermore, transcript analysis using primers common for all three isoforms of murine CD24 showed at least 16-fold higher mRNA expression in Susp cells as compared to Adh cells (Fig. 4).

Next, we genetically labeled the cell membrane and cytoplasm of B16-F10 cells by expressing a membrane-targeted HA-tag, and a cytoplasm-targeted clover fluorescent protein under a constitutive promoter. This was done to determine the general pattern of membrane protein expression in the Susp subpopulation. The HA-tag on the cell surface was detected using flow cytometry analysis with an HA-tag specific monoclonal antibody (clone 16B12). Approximately 88% of the Susp subpopulation stained positive for HA-tag expression on the cell surface, with no expression of the cytoplasmic marker clover.

**Fig. 5:**
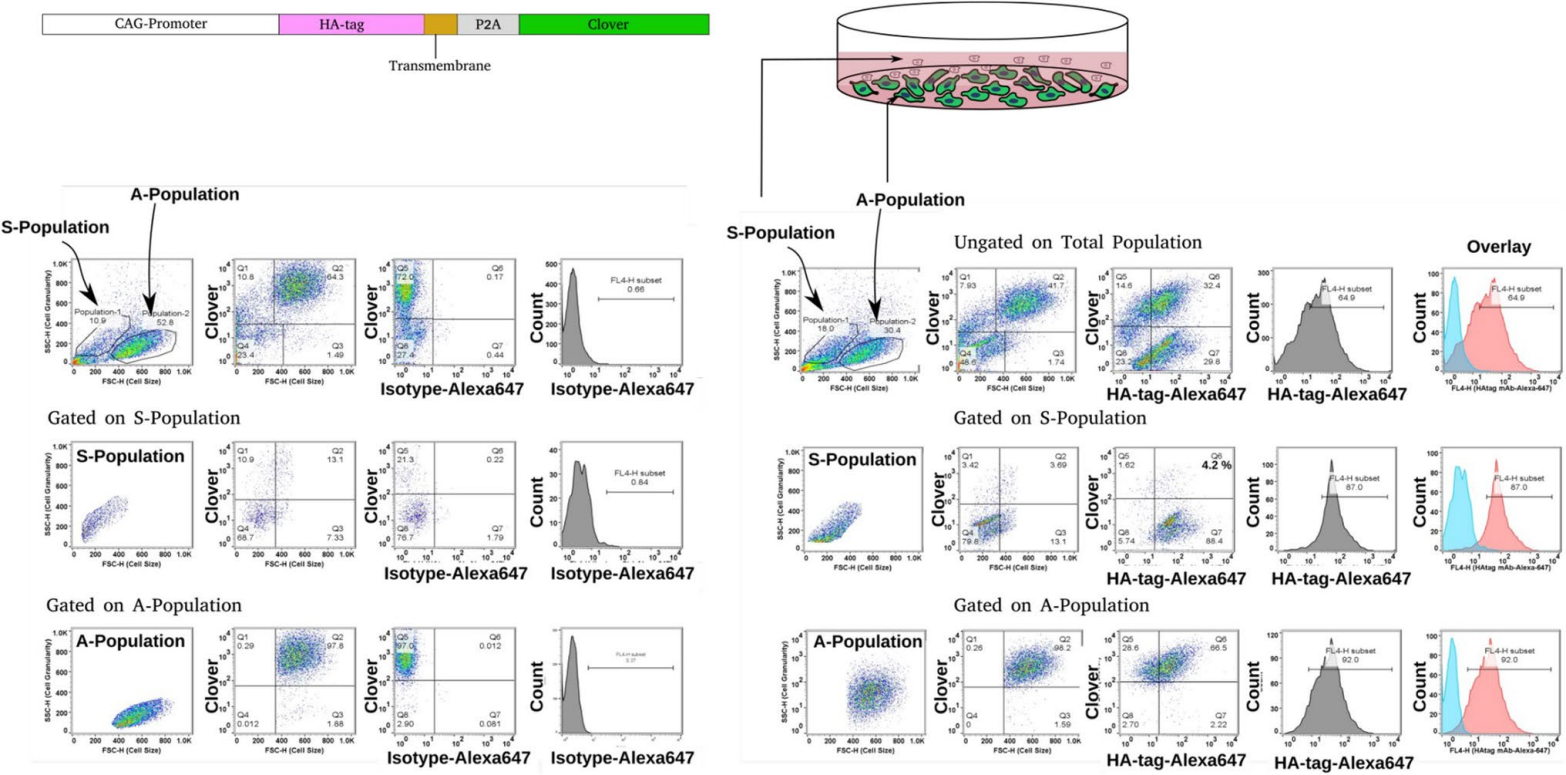
**Visualizing cytoplasmic and transmembrane protein expression in Susp subpopulation.** SB-Transposon-based expression construct used for generating B16-F10-tmHA cell lines that express cytoplasmic Clover fluorescent protein and cell surface-expressed transmembrane HA-tag (tmHA), detectable by flow cytometry-compatible HA-tag mAbs. Susp (S) and Adh (A) subpopulations identified in cell cultures are depicted. Flow cytometry dot plots showing expression of clover and tmHA in ungated total cells and gated subpopulations. Isotype control staining is shown in the left panel of the figures, and CD24-specific staining is shown in the right panel of the figures

On the other hand, only about 2% of the Adh subpopulation, which lacks the cytoplasmic marker, showed positivity for cell surface expression of the HA-tag. Additionally, only about 4.2% of the Susp subpopulations stained positive for both the cytoplasmic marker and the cell surface marker. While 93% of the Adh subpopulations were positive for the cytoplasmic marker, only about 66% were positive for both the cytoplasmic and cell surface markers. This means that approximately 27% of the Adh subpopulation expressed only the cytoplasmic marker with no expression of the cell surface HA-tag (Fig. 5).

**Fig. 6:**
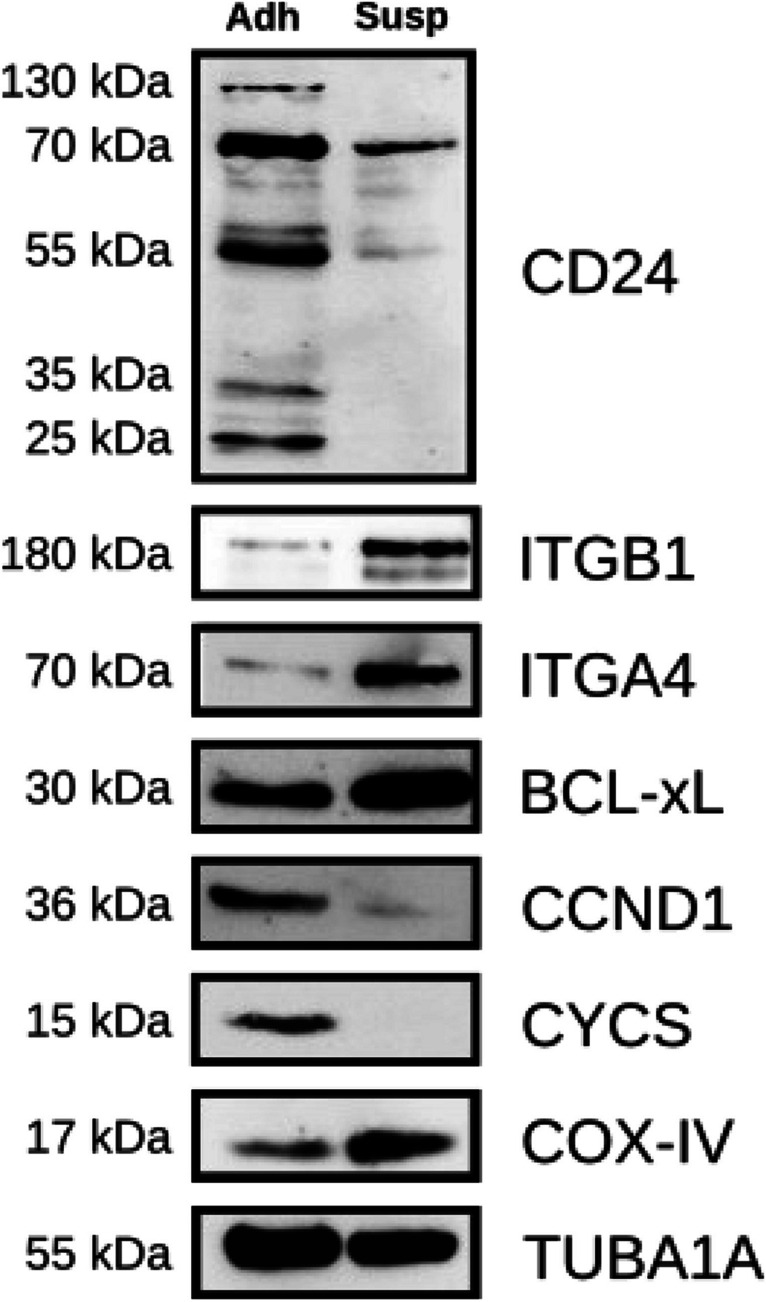
**Total protein expression analysis of Adh and Susp subpopulations.** Differential protein expression in Adh and Susp subpopulations isolated from confluent B16-F10 cell cultures. The immunoblot analysis highlights the expression levels of total CD24, along with several proteins associated with adhesion, metabolism, survival, and proliferation. α-Tubulin (TUBA1A) serves as the internal control for normalization

Total protein expression analysis by immunoblotting of the total cell lysates of the Adh and Susp subpopulations further consolidated the differential expression levels of CD24 among the B16-F10 subpopulations, as bands of different sizes were detected in both the Adh and Susp subpopulations. CD24 levels were considerably higher in the Adh cells, with the most prominent signal intensities observed at 70 and 55 kDa, respectively (Fig. 6). In addition, the Adh subpopulation was characterized by the presence of a CD24 band with a considerably high molecular weight of about 130 kDa, suggestive of heavily glycosylated forms of CD24 [22]. The Susp cells lacked some of the bands otherwise detectable in the Adh subpopulation and showed an overall decrease in protein expression, with the highest signal intensity detected at 70 kDa. In addition, we examined the expression levels of integrin α4β1. Western blot analysis showed that the α4 (ITGA4) and β1 (ITGB1) integrin subunits exhibit different expression patterns in the Adh and Susp B16-F10 subpopulations. The heterodimer was upregulated in the Susp cells, where strong bands were observed for both of its subunits. On the other hand, α4β1-integrin was significantly reduced, if not completely absent, in the Adh cells. Taken together, Susp cells reflect FSC^−ve^SSC^+ve^ cells with a unique expression pattern for CD24. Our analysis of cell surface and intracellular CD24 expression revealed unique expression patterns not previously reported.

### CD24-enriched subpopulation express markers of apoptosis

**Fig. 7:**
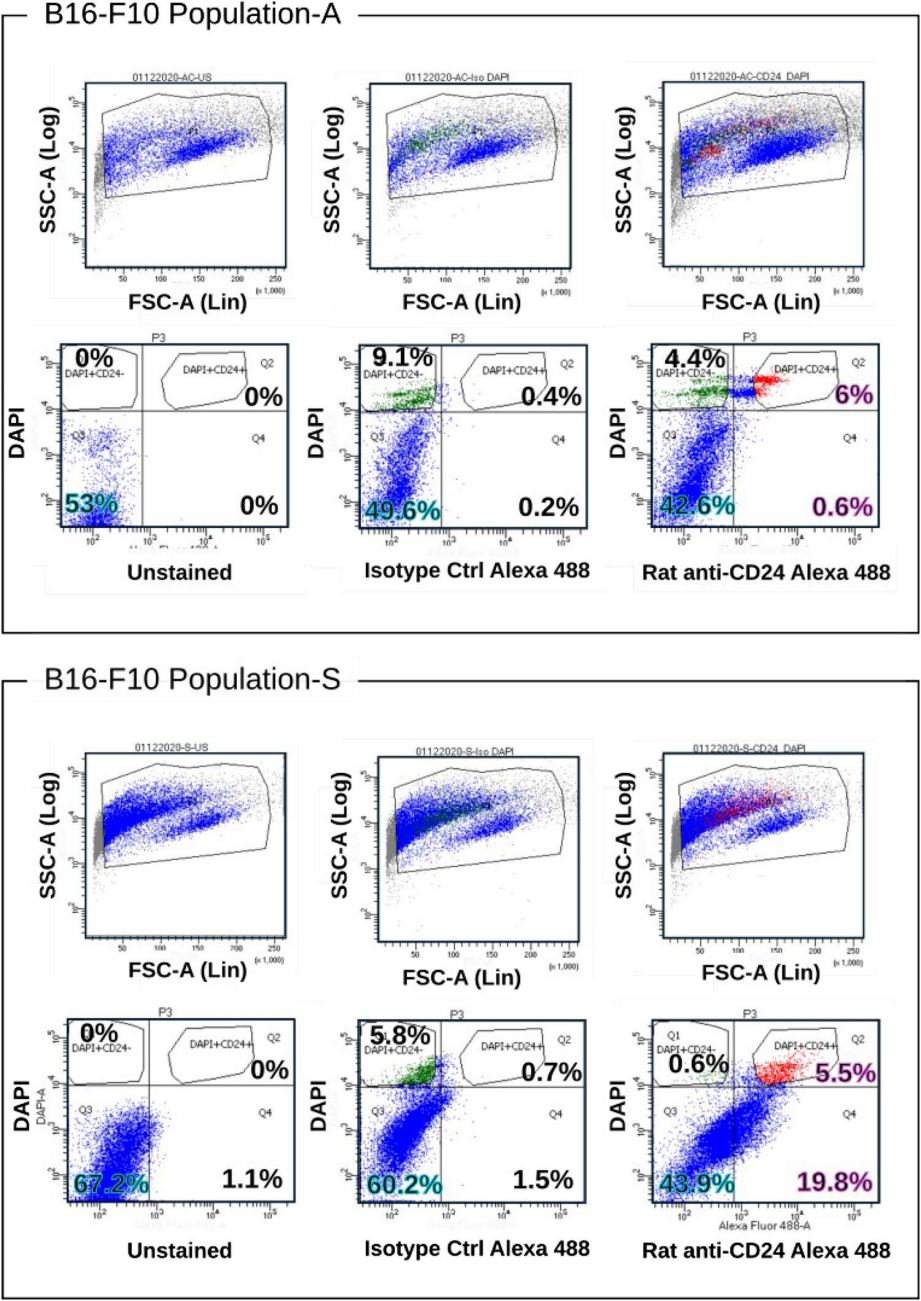
**Flow cytometry staining for DAPI and CD24.** DAPI staining for Adh and Susp B16-F10 cells. Top panels show forward and sideward scatter plots. Bottom panels show double staining for DAPI and CD24. For both Adh and Susp cells, the first column represents an unstained sample, the second column – rat isotype control staining, and the third column shows a rat anti-CD24 staining. Around 10% of the Adh and 6% of the Susp cells are double positive for DAPI and CD24

**Fig. 8:**
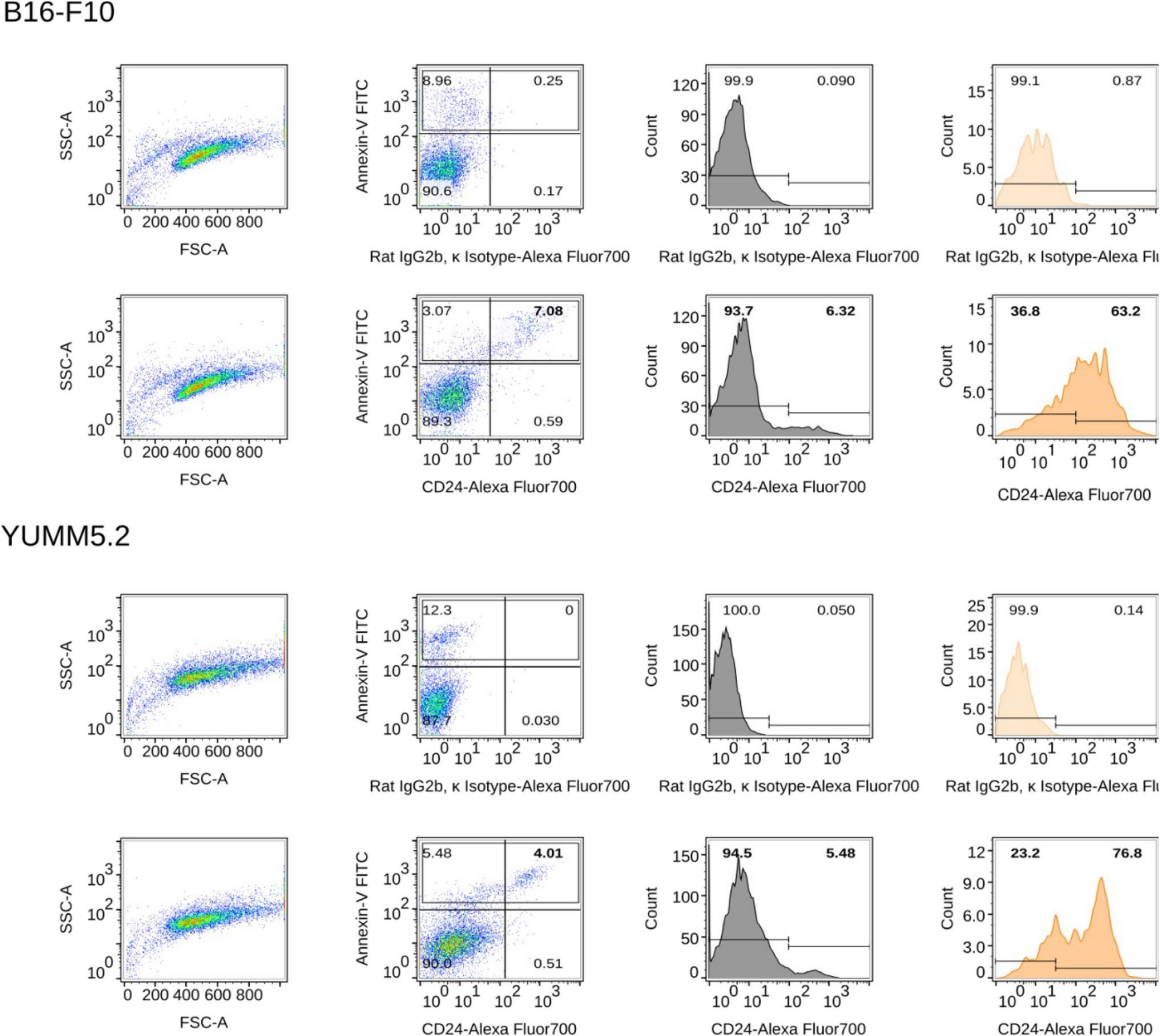
**Surface expression analysis of CD24 and apoptosis markers in melanoma cells.** Flow cytometry analysis showing combined staining for the apoptosis marker Annexin-V and CD24 in two different melanoma cell lines. Unstained and isotype staining controls were used to quantify double positive populations

Dead cells and cell debris floating over the adherent cells generally have reduced light scattering and could be attributed to Susp cells. Furthermore, Susp cells displayed fragmented DNA bands on agarose gel electrophoresis at the genomic DNA level, whereas the Adh subpopulation did not (Supplementary Fig. 5). This result was further corroborated by evaluating the percentage of dead cells by a 4',6-diamidino-2-phenylindole (DAPI) staining of the B16-F10 cells. This staining showed that around 10% of the Adh and 6% of the Susp B16-F10 cells were positive for DAPI and, thus, were considered to be dying (Fig. 7). In both subpopulations, approximately 6% of the cells were positive for both CD24 and DAPI. Whereas almost none of the Adh cells that were DAPI-negative revealed surface expression of CD24, about 20% of DAPI-negative Susp cells stained positive for CD24. Furthermore, double staining for Annexin V and CD24 showed that more than 90% of Susp cells were positive for Annexin V, and about 6% showed positivity for both Annexin V and CD24 in B16-F10 cells. In contrast, only about 15% of Adh cells stained positive for Annexin V, and about 3% showed positivity for both Annexin V and CD24. Neither the Susp nor Adh populations contained CD24^+ve^ cells that were Annexin V-negative (Fig. 8, Supplementary Fig. 6). Similarly, more than 85% of Susp cells in YUMM5.2 cultures were positive for Annexin V, and more than 50% showed positivity for both Annexin V and CD24 (Fig. 8, Supplementary Fig. 7). Taken together, the cell surface expression of CD24 is specifically confined to a subpopulation demonstrating the onset of apoptosis, characterized by phosphatidylserine externalization (phosphatidyl membrane flipping) detected by Annexin-V staining, and nuclear permeability to propidium iodide.

### Tumorigenic properties of CD24-enriched Susp cells

**Fig. 9:**
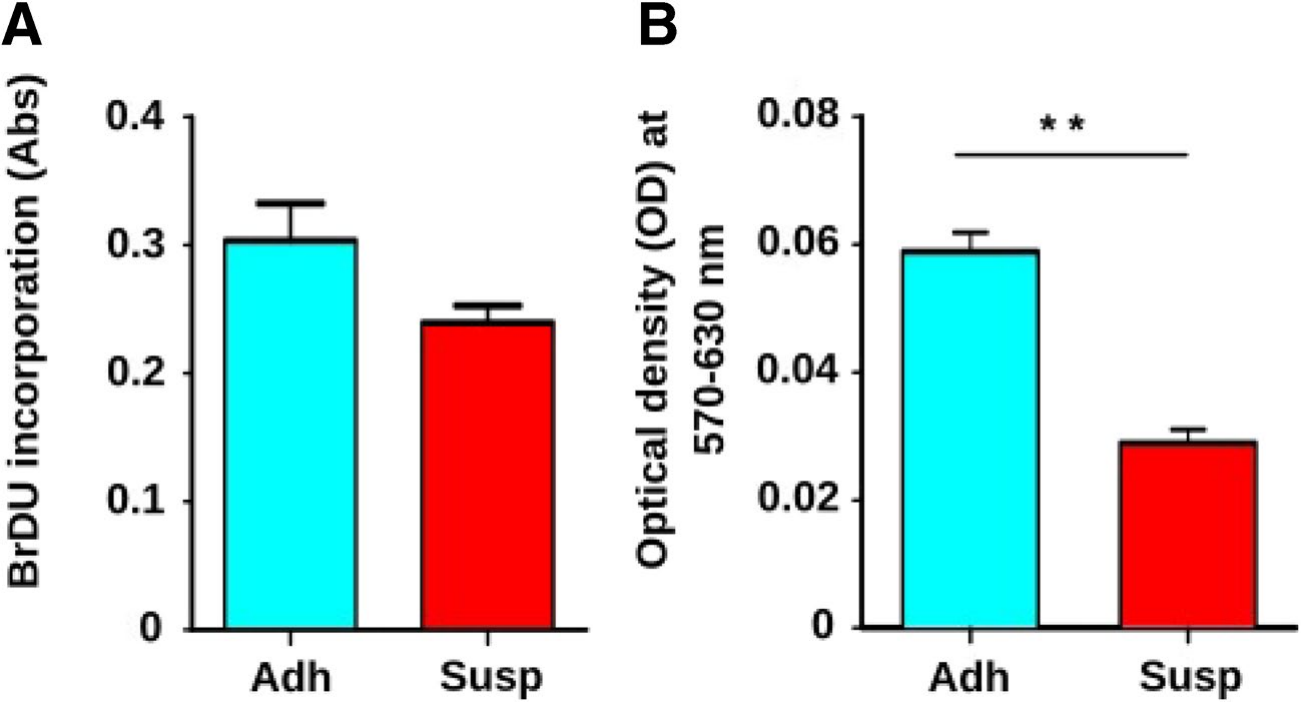
**Proliferation and metabolic activity of Susp subpopulation.** (**A**) Assessment of BrdU incorporation as a measure of proliferation properties of Susp (red) and Adh subpopulations in in vitro cultivated B16-F10 melanoma cells. (**B**) Assessment of MTT formation as a measure of mitochondrial metabolic activity of Susp and Adh subpopulations in vitro cultivated B16-F10 melanoma cells

**Fig. 10:**
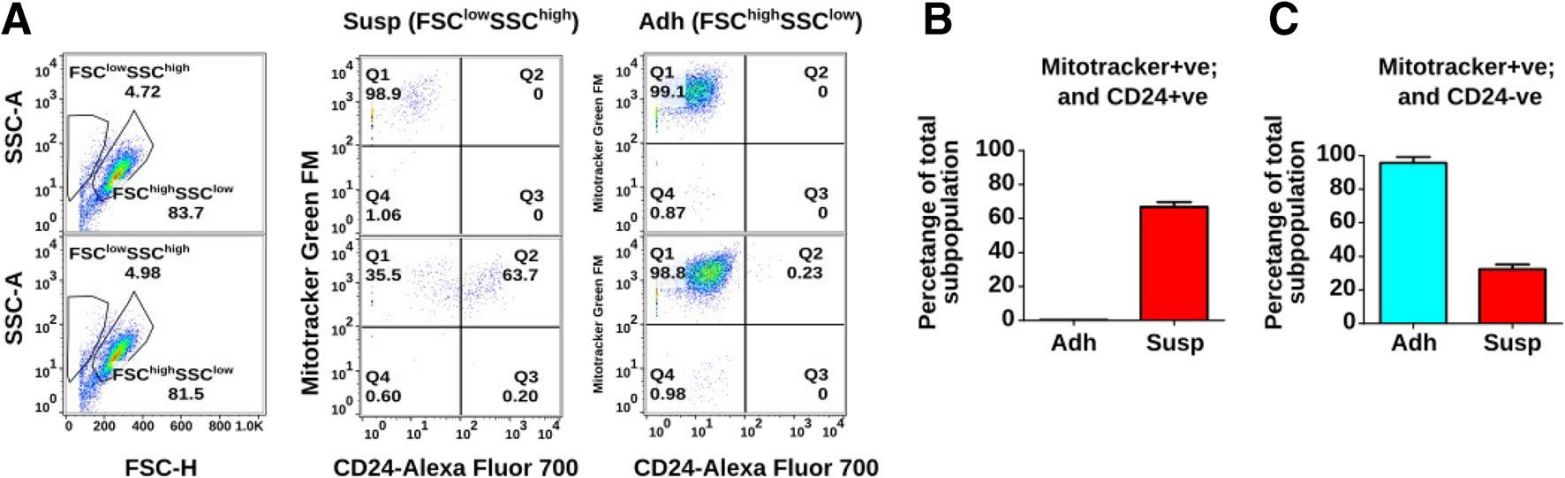
**Presence of mitochondria in Susp Cells.** (**A**) Assessment of mitochondria in Susp and Adh subpopulations of B16-F10 melanoma cells. (**B**) Assessment of MTT formation as a measure of mitochondrial metabolic activity of Susp and Adh subpopulations in vitro cultivated B16-F10 melanoma cells

**Fig. 11:**
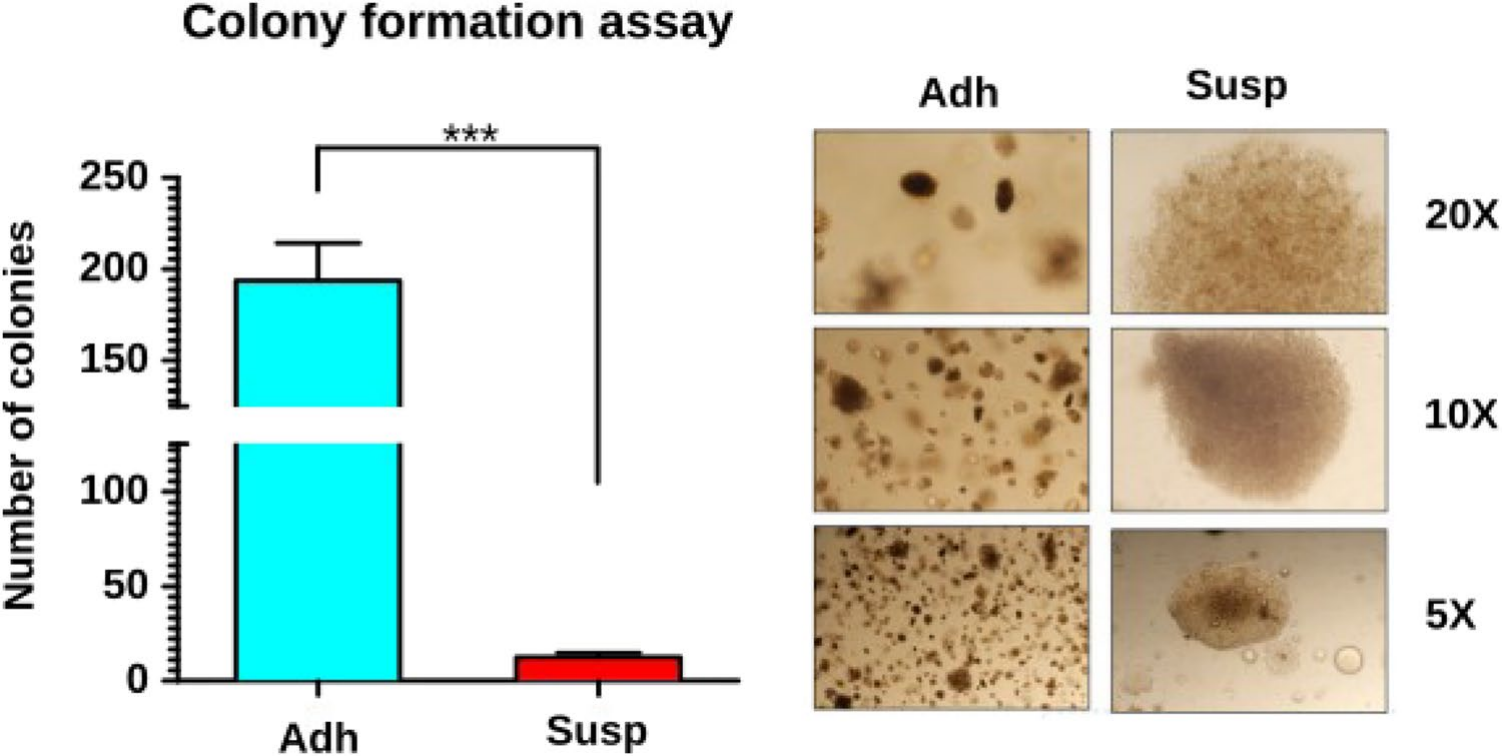
**Soft agarose 3D colony formation assay. **Assessment of anchorage independent growth properties of Susp and Adh subpopulations. Sorted subpopulations were counted regardless of Trypan blue positivity and equal numbers were inoculated in soft agarose and colony formation propensities were measured 3-weeks after inoculation. Representative images of colonies formed are shown

**Fig. 12:**
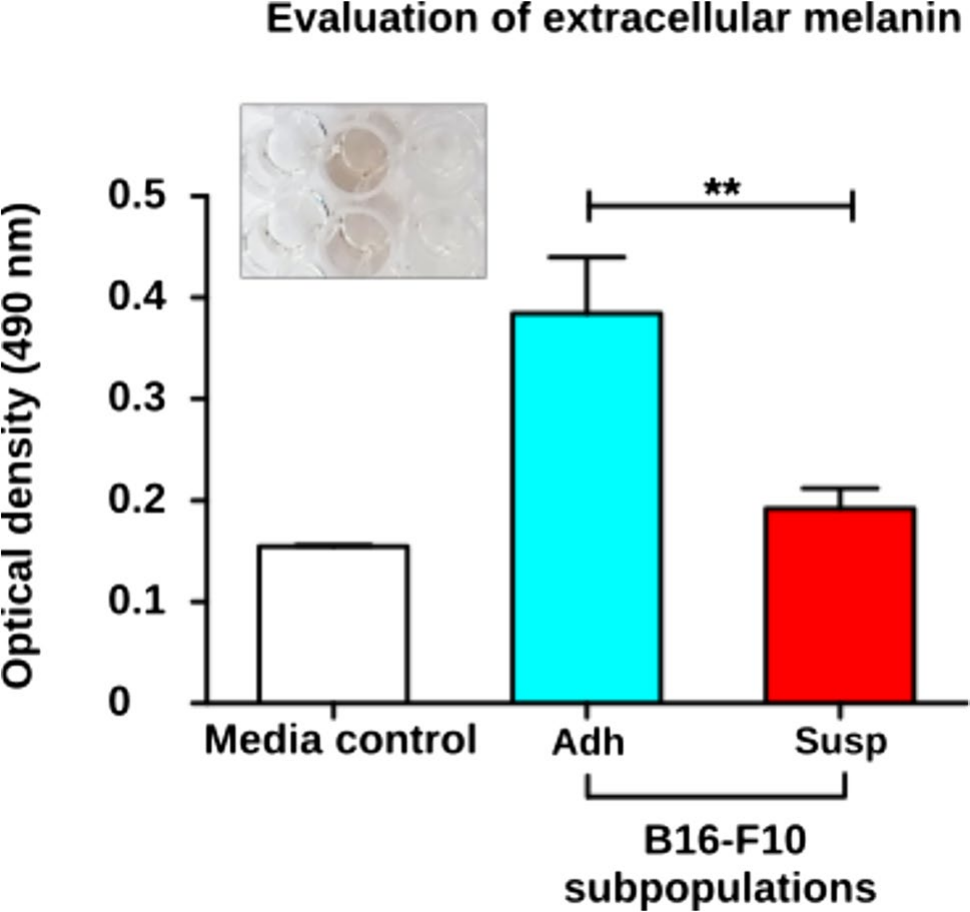
**Quantification of secreted pigment in 3D culture supernatants.** Assessment of pigment synthesizing potential of Susp and Adh subpopulations of B16-F10 cells by spectrophotometric measurement of the optical density of the culture supernatants from the 3D colony formation assay where cells were cultivated in dye-free complete RPMI medium. Inset shows images of the culture supernatant from the media control, Adh, and Susp 3D culture, respectively

Although more than 90% of Susp cells were positive for Annexin V, with about 6.5% being positive for both Annexin V and CD24, Susp cells unexpectedly showed a clearly detectable BrdU incorporation comparable to Adh cells, which consist of about 90% Annexin V-negative living cells (Fig. 9A). To determine if Susp cells are metabolically active, mitochondrial oxidoreductase activities were quantified using the 3-[4, 5-dimethylthiazol-2-yl]-2,5-diphenyltetrazolium bromide (MTT) assay. A two-fold reduction in mitochondrial activity was observed in Susp cells, suggesting a low but intact mitochondrial function (Fig. 9B). The presence of mitochondria in Susp cells was confirmed by flow cytometry staining using MitoTracker™ Green FM (Fig. 10A). Over half of all Susp cells contained mitochondria (Fig. 10B), while the entire Adh subpopulation stained positive for mitochondria (Fig. 10C). Furthermore, immunoblot analysis indicated a significant difference in the expression of inner mitochondrial proteins between Susp and Adh cells, with cytochrome c protein being absent in Susp cells and COX IV proteins being upregulated, as compared to Adh cells (Fig. 6). Interestingly, the outer mitochondrial anti-apoptotic protein BCL-XL was upregulated in Susp cells when compared to Adh cells (Fig. 6). In addition to metabolism related proteins, Susp cells also showed expression of cell cycle promoting genes. For instance, detectable expression of CCND1 was observed in Susp cells, albeit at lower levels compared to Adh cells. Concordantly, anchorage-independent growth assay revealed that Susp cells possessed the ability to form colonies, and it also indicated qualitative and quantitative differences between the colonies formed by Susp and Adh cells. While the Adh cells formed numerous colonies, their growth was likely stopped after reaching the saturation phase. In contrast, the Susp cells formed fewer, but larger colonies, indicative of uninterrupted continuous growth. (Fig. 11). Interestingly, nearly all colonies formed by Susp cells lacked melanin, as inferred from the measured levels of secreted pigment in the culture supernatants, potentially suggesting a less differentiated state. These results indicate CD24 + ve Susp cells do not progress into the completion of apoptosis, rather strangely retain potential to proliferate. Accordingly, we found CD24 + ve Susp growing in the anchorage independent growth in 3D low melt agarose supplemented with complete growth medium (Fig. 12).

## Discussion

Anastasis is a process where seemingly apoptotic cells can recover and resume their normal cellular activities or proliferation under certain conditions. In the present study, we investigated the ability of seemingly apoptotic metastatic melanoma cells to grow colonies in the absence of external anchorage signals, thus providing the first evidence for anastasis in a murine melanoma cell line. Using B16-F10 as the cancer cell model, we explored the tumorigenic properties of apoptotic and granular CD24^+ve^FSC^low^ cells, which are actively shed in the exponentially growing metastatic melanoma cell lines.

We found a heterogeneous expression pattern for CD24 in B16-F10 cells, with cell surface expression detected exclusively in the FSC^low^SSC^high^ Susp population. The presence of subpopulations in B16-F10 cells that express CD24 exclusively on the cell surface of FSC^low^SSC^high^ is intriguing. By contrast, intracellular expression was detected in both Susp and Adh cells. Susp cells seem to contain the mature form of CD24 on their surface, as only a limited level of cytoplasmic CD24 expression could be detected by immunoblot and intracellular flow cytometry staining. Despite lower cytoplasmic expression of CD24 proteins in Susp cells, mRNA expression was about 16-fold higher as compared to Adh cells. The discrepancy between lower cytoplasmic protein expression and high cell surface expression of CD24 could be due to several reasons. It is possible that the GPI anchor biosynthesis for CD24 is very efficient leading to rapid transport of the protein to the cell surface. Additionally, it is also possible that CD24 has a longer half-life leading to accumulation at the cell surface despite lower cytoplasmic expression. Another possibility is that the regulation of CD24 expression occurs primarily at the post-transcriptional level, such as by modulation of protein degradation or trafficking, rather than solely at the transcriptional level. This could result in a situation where high mRNA expression of CD24 does not necessarily correlate with high cytoplasmic protein expression but does lead to high cell surface expression. In any case, the mechanisms governing the expression and regulation of GPI-anchored proteins, including CD24, are complex and multifaceted [23–25], which points to the need for further pertinent studies.

When it comes to cytoplasmic CD24, several reports suggest contradicting roles for cytoplasmic CD24 in tumor progression. In pancreatic cancer, knockdown of cytoplasmic CD24 enhances tumor progression by increasing invasiveness and liver metastasis [26]. In other cancer cell line models, however, depletion of cytoplasmic CD24 enhanced apoptosis [27, 28]; and in a murine model of hepatocellular carcinoma, overexpression of cytoplasmic CD24 contributes to p53-dependent cell cycle progression [29]. Similarly, contradicting information regarding the surface expression of CD24 can be found. Both CD24^+ve^ [29, 30] and CD24^−ve^ [31, 32] subpopulations are considered to possess self-renewal properties in various cancer types, including melanoma [33], and are therefore widely used as a marker to isolate cancer stem cells. However, we strongly believe that our findings might have unraveled a novel mechanism that may have been overlooked or gone unidentified previously and could potentially explain the discrepancies observed in various studies.

Generally, the FSC^low^SSC^high^ quadrant in flow cytometry analysis settings is considered an area where dying and nonviable subpopulations appear [34]. Susp cells, which are the population of floating cells over the surface of B16-F10 monolayers, appear in the FSC^low^SSC^high^ quadrant. Expectedly, we found that about 90% of FSC^low^SSC^high^ Susp cells were apoptotic, as indicated by the membrane flipping [35] detected by Annexin V staining. About 6% of the FSC^low^SSC^high^ Susp population was positive both for CD24 and Annexin V, and the same percentage was also positive both for CD24 and DAPI. None of the FSC^low^SSC^high^ Susp subpopulations of B16-F10 that stained negative for Annexin V contained CD24 on the cell surface. We were able to largely rule out a potential non-specific binding or staining artifacts since all CD24 surface stainings performed were compared to the corresponding isotype-matched stainings. Although CD24^+ve^ Susp cells expressed bonafide markers for apoptosis, metabolic activity was still intact. It is known that apoptotic cells, to some degree, can retain plasma membrane integrity and metabolic activity as the process proceeds to completion [36]. Accordingly, we detected mitochondrial activity in FSC^low^SSC^high^CD24^+ve^ Susp cells. Furthermore, in about 20% of FSC^low^SSC^high^CD24^+ve^ Susp cells, the integrity of the cell membrane and nucleus was preserved, as indicated by the lack of DAPI positivity. FSC^low^SSC^high^CD24^+ve^ Susp cells lacked cytochrome c and contained higher amounts of BCL-XL, which is a potential molecular mechanism by which Susp cells resist apoptosis [37] and promote survival [38]. This dysregulated protein expression pattern could indicate a unique survival advantage for the Susp population. BCL-XL is an anti-apoptotic protein that inhibits apoptosis, while COXIV is a subunit of the electron transport chain, which generates energy for the cell. Elevated expression of these proteins could promote cell survival by preventing apoptosis and enhancing energy production. On the other hand, undetectable expression of CCND1 and lower expression of CYCS could indicate that the cell is not actively proliferating and is in a quiescent or non-dividing state. CCND1 is a protein involved in cell cycle regulation, specifically the G1 to S phase transition, and its absence could indicate a lack of cell division. CYCS is a protein involved in apoptosis, and its absence could indicate a reduced susceptibility to programmed cell death. Taken together, these findings suggest that the cell may have an advantage in surviving and maintaining its current state rather than actively dividing. The dysregulated expression of BCL-XL and COXIV may allow the cell to maintain energy production and prevent cell death, while the absence of CCND1 and CYCS may indicate a lack of cell division and reduced susceptibility to apoptosis.

Although FSC^low^SSC^high^CD24^+ve^ Susp cells retain plasma membrane integrity and, to a certain degree, metabolic activity, genomic DNA appears to exhibit features of fragmentation, which is considered a hallmark of the late stage of apoptosis [39]. Intriguingly, FSC^low^SSC^high^CD24^+ve^ Susp cells that were trypan blue positive when inoculated in soft agarose medium recovered despite DNA fragmentation and grew into large colonies under 3D culture conditions.

It is possible for cells to survive and proliferate despite having fragmented genomic DNA under certain conditions. One possible explanation for this phenomenon is that the cells are undergoing a form of programmed cell death called anoikis, which occurs when cells detach from the extracellular matrix (ECM) and lose their attachment to neighboring cells. Anoikis can lead to the fragmentation of genomic DNA [40]. But some cells can still survive and proliferate if they are able to adapt to the 3D culture conditions and establish new cell–cell and cell-ECM interactions [41–43]. Another possible explanation is that the cells have activated DNA repair mechanisms that are able to repair the fragmented DNA and prevent cell death [44]. This could be due to the dysregulated mitochondrial protein expression pattern mentioned earlier, which may have activated DNA repair pathways in the cells [45]. Cells can recover from apoptosis after DNA damage has occurred. Therefore, we do not rule out the possibility of FSC^low^SSC^high^CD24^+ve^ Susp cells recovering from late-stage apoptosis via a process that possibly leads to chromothripsis [9, 46]. Further studies investigating the chromosomal integrity of colonies formed by Susp cells can validate these speculations. Overriding of the apoptosis program by seemingly apoptotic FSC^low^SSC^high^CD24^+ve^ Susp cells to recover even after having progressed to the nuclear DNA fragmentation step is very likely a characteristic feature of malignant cells undergoing anastasis and acquiring resilience.

While it is unusual for cells with fragmented genomic DNA to survive and proliferate, it is quite possible under certain conditions, such as 3D culture conditions in cells that lack the molecular program for apoptosis but have intact machinery for the activation of DNA repair mechanisms. Further investigations are needed in support of our findings and to determine the precise mechanism underlying the survival and proliferation of CD24^+ve^Annexin^+ve^ FSC^low^SSC^high^ melanoma cells.

Taken together, our findings suggest that seemingly apoptotic metastatic melanoma cell lines express CD24 and possess tumorigenic properties in vitro. Therefore, we conclude CD24 as a novel cell surface marker for anastasis in malignant melanoma cells.

Anastasis-prone CD24^+ve^ subpopulations that are floating and small and exhibit all the surface features of dying cells are better suited to spread in poorly vascularized tissues, escape detection from immune cells, overwhelm or misguide cancer-fighting immune cells, thereby contributing to the metastatic melanoma progression. It has not escaped our notice that CD24^+ve^ subpopulations, which also express high levels of PD-L1, may potentially contribute to inhibitory signaling in adaptive immune responses. We are currently exploring this hypothesis to investigate whether the FSC^low^SSC^high^CD24^+ve^ subpopulation interacts with naive T cells and modulate the antitumoral T cell response against the FSC^high^SSC^low^CD24^−ve^ subpopulation of melanoma cells.

## Methods

### Cell lines

C57BL/6-derived mouse metastatic melanoma cell line B16-F10 (ATCC® CCL-6475™) was obtained from the American Type Culture Collection (ATCC) and YUMM5.2 was a generous gift from Bettina Weigelin's lab. Cells were cultivated (grown) in complete RPMI 1640 medium with 10% fetal calf serum, 1% nonessential amino acids (NEAA), 1% L-Glutamine and 1% Penicillin–Streptomycin. Cells were regularly passaged upon reaching a confluency of around 80%. Throughout the process, the two B16-F10 subpopulations – adherent (hereafter referred to as Adh) and suspension (hereafter referred to as Susp) were handled separately. For this purpose, the culture medium containing the Susp cells was collected and centrifuged for 5 min at 300 RCF. The cell pellet was subsequently resuspended in 1xPBS (Phosphate-Buffered Saline) and kept on ice. Meanwhile, the remaining adherent monolayer was rinsed with 1xPBS and treated with 1 mM PBS/EDTA. Following detachment, cells were centrifuged for 5 min at 300 RCF and resuspended in the corresponding growth medium. A small fraction of the Adh and Susp cells was collected for experimental purposes, while the rest was used for maintenance of the cell line. The respective number of cells was seeded on T175 flasks and kept under humidified conditions at 37 °C with 5% CO_2_. Periodically, PBS-washed Adh and Susp cells were collected and stored as cell pellets at -80 °C for future usage. Cell lines used in this study were on the International Cell Line Authentication Committee list of currently known cross-contaminated or misidentified cell lines. The mouse cell lines used in this study were confirmed to be free of any mycoplasma contamination.

### Flow cytometry staining

B16-F10 subpopulation cells were washed in ice-cold PBS and resuspended in FACS staining buffer (PBS + 2% FCS + 0.25% NaN3) and incubated for 10 min on ice. Antibodies (Abs) and isotype control IgGs were added to the cell suspension to a dilution of 1:50 to 1:100 and incubated further for 1 h on ice. After the primary antibody incubation, the stained cells were washed once in FACS staining buffer and once in normal PBS prior to further analysis.

For cell surface staining, the following Abs were used:

Alexa Fluor® 700 Rat Anti-Mouse CD24, Clone M1/69 (RUO) (BD Pharmingen™, #564237), Alexa Fluor® 700 Rat IgG2b, κ Isotype Control (BD Pharmingen™, #557964), PerCP/Cyanine5.5 anti-mouse CD24 Antibody (BioLegend, #101823), PerCP/Cyanine5.5 Rat IgG2b, κ Isotype Ctrl Antibody (BioLegend, #400631); Brilliant Violet 510™ anti-mouse CD24 Antibody (BioLegend, #101831), Brilliant Violet 510™ Rat IgG2b, κ Isotype Ctrl Antibody (BioLegend, #400645); PE/Cyanine7 anti-mouse CD274 (B7-H1, PD-L1) Antibody (BioLegend, #124313), PE/Cyanine7 Rat IgG2b, κ Isotype Ctrl Antibody (BioLegend, #400617); PE anti-mouse CD83 Antibody (BioLegend, #121507), PE Rat IgG1, κ Isotype Ctrl Antibody (BioLegend, #400407); Alexa Fluor® 647 anti-HA.11 Epitope Tag Antibody (BioLegend, #682404), Alexa Fluor® 647 Mouse IgG1, κ Isotype Ctrl (ICFC) Antibody (BioLegend, #400135).

For combined cell surface and intracellular staining, cells were washed once in PBS followed by staining for CD24 using anti-CD24 IgG2a (Biozol GmbH, #SBA-1590–09) and IgG2a rat (Thermo Fisher Scientific, #R58788) as isotype control by following the cell surface staining protocol described above. After the end of cell surface staining, PBS-washed cells were fixed in 4% PFA at RT for 15 min. Fixed and washed cells were permeabilized using 0.03% Triton X 100 in PBS for 30 min at room temperature (RT). Permeabilized cells were incubated in Rabbit anti-CD24 Ab (Abcam, #ab175088) and Rabbit IgG isotype control for 1 h at RT in the dark. After the completion of primary Ab incubation, fluorochrome conjugated secondary anti-rabbit Ab in permeabilization buffer was added and incubated for 45 min at RT in dark, followed by washing and resuspension in PBS.

For Annexin V staining, PBS washed cells were washed once in 1X Annexin V binding buffer (BD Biosciences, #556,454) and stained with Annexin V-FITC (BioLegend, #640,905) at a dilution of 1:100 at RT in the dark on ice. After 15 min, cells were washed twice in 1X Annexin V binding buffer and once in PBS before resuspension in PBS.

### Mitochondria staining

Mitochondria staining was performed using Mitotracker Green (Invitrogen-Thermo Fischer, #M7514). A working solution of Mitotracker Green was prepared by diluting the stock concentration (744.2 μM) to a final concentration of 40 nM in phosphate-buffered saline (PBS). Approximately 0.5 to 1 million cells were resuspended in 1 mL of pre-warmed PBS. To this cell suspension, 40 nM of the Mitotracker Green working solution was added, and the cells were then incubated at 37 °C for 15 min to allow for mitochondrial staining. Following the incubation period, the stained cells were centrifuged to pellet them, and the supernatant was discarded. The cell pellet was washed once with large volumes of pre-warmed PBS to remove excess Mitotracker Green and then resuspended in FACS PBS for flow cytometry analysis. Adjustments to cell concentration, staining duration, and other parameters were made based on cell types.

### Plasmids and transfection

Plasmid expression constructs with coding DNA sequences for clover fluorescent protein and bicistronic self-cleavable P2A peptide separated clover fluorescent protein and HA-tag fused to CD8a transmembrane segment were cloned into a sleeping beauty plasmid backbone vector as previously described [47].

To generate stable cell lines, plasmid DNA constructs were mixed with a plasmid encoding SB transposase B16-F10 cells using Lipofectamine 2000 (Thermo Fisher Scientific, #11,668,019) according to the manufacturer’s instructions. Transfected cell lines were selected first by puromycin treatment and finally by flow cytometry-based cell sorting of clover positive B16-F10 cells.

### Western blot

Freshly isolated or thawed cell pellets were dissolved in a lysis buffer and kept on ice for half an hour while being resuspended every 10 min. Following incubation, lysates were centrifuged at approx. 14 000 rpm for 10 min at 4 °C. The supernatant was then collected into new tubes and, if not immediately utilized, it was stored at -20 °C. In order to determine the protein concentration, 1 μL of each lysate was mixed with 1 ml of 1:5 diluted Protein Assay Dye Reagent Concentrate (Bio-Rad, Hercules, California, USA). Samples were left to incubate for 10 min at RT, after which the concentration was derived by measuring the absorbance at 595 nm using the Genesis 10 Biophotometer (Thermo Fisher Scientific, Waltham, Massachusetts, USA).

Immunoblot analysis was performed on whole cell lysates. For this purpose, a total protein amount of 50 μg/μl was used. To achieve this concentration, samples were firstly diluted with lysis buffer and subsequently with 5xLaemmli to ensure proper denaturing conditions. Samples were left to incubate at 37 °C for 15 min, after which they were loaded on either a 10% or 15% SDS polyacrylamide gel. To monitor the molecular weight, PageRuler Plus Prestained Protein Ladder (Thermo Fisher Scientific, Waltham, Massachusetts, USA) was used. Subsequently, electrophoresis was conducted with the Protean 3 Chamber (BioRad, Hercules, California, USA) filled with an SDS-PAGE running buffer. Protein samples were left to resolve at 120 V for approximately 2 h. Following completion, they were electro transferred on nitrocellulose membranes using the Trans-blot semi-dry transfer system (BioRad, Hercules, California, USA). Afterwards, membranes were incubated with a stripping buffer for 15 min at 55 °C and were subsequently washed twice with TBST for approx. 10 min each. To avoid non-specific antibody binding, membranes were blocked in TBST with 5% (w/v) nonfat dry milk powder for 1 h at RT. After the blocking step, primary antibodies were incubated overnight at 4 °C. On the next day, membranes were washed 3 times for 10 min each with TBST before being incubated with horseradish peroxidase (HRP) conjugated secondary antibodies. Prior to visualization, membranes were covered with Luminata TM Western HRP Substrates solution (PAN-Biotech GmbH, Aidenbach, Germany) and developed with the LAS 4000 Imager (GE Healthcare, Chicago, Illinois, USA). Multiple exposures were taken to select images within the dynamic range of the digital Imager.

The following Abs were used for immunoblotting analyses:

Anti-CD24 mAb Rabbit (Proteintech, St. LeonRot, Germany GMBH, 10600–1-AP), Anti-COX-IV pAb Rabbit (Proteintech, St. LeonRot, Germany GMBH 11242–1-AP), Anti-CYCS mAb Mouse (BD Pharmingen, San Diego, California #556432), Anti-TUBA1A mAb Rabbit (Cell Signaling Technology, Danvers, Massachusetts #2125), Anti-CCND1 mAb Rabbit (Cell Signaling Technology, Danvers, Massachusetts, USA #2978), Anti-Bcl-X(L) mAb Rabbit (Cell Signaling Technology, Danvers, Massachusetts, USA #2764), Anti-ITGB1 mAb Rabbit (Cell Signaling Technology, Danvers, Massachusetts, USA #34971), Anti-ITGA4 mAb Rabbit (Cell Signaling Technology, Danvers, Massachusetts, USA #8440), HRP-linked anti-mouse IgG Goat (Promega GmbH, Walldorf, Germany #W4011) and HRP-linked anti-mouse IgG Goat Promega GmbH, Walldorf, Germany, # PA1-74421).

### Immunohistochemistry

Tissues were fixed overnight in 4% paraformaldehyde in PBS (pH 7.4) at 4 °C. Fixed tissues were embedded in paraffin and sliced. Sections were prepared for staining first by deparaffinization followed by hydration in the following solutions: 3 washes of xylene 5 min each, two washes of 100% ethanol 10 min each, two washes of 95% ethanol 10 min each and two washes in distilled water 5 min each. Antigen retrieval was obtained by incubation with a heated citrate buffer (sodium citrate 10 mM, pH 6) for 15 min. Immunohistochemistry was performed as per our standard procedures. Briefly, after antigen retrieval sections were incubated with 3% hydrogen peroxide for 10 min to quench endogenous peroxidase activity. Non-specific background staining was blocked by incubating in UltraVision Block (Thermo Scientific, # TA-060-PBQ) for 5 min at room temperature. CD24 staining was done by incubating in rabbit anti-CD24 mAb Anti-CD24 mAb Rabbit (Proteintech, St. LeonRot, Germany GMBH, Cat No. 10600–1-AP) at a dilution of 1: 400 overnight at 4° C. For isotype control staining Rabbit IgG (Proteintech, St. LeonRot, Germany GMBH, Cat No. 30000–0-AP) was used. Detection was achieved using HRP Polymer (Thermo Scientific, # TL-060-PH) followed by incubation with peroxidase compatible DAB chromogen.

### Reverse transcriptase PCR

Total RNA was extracted from freshly isolated or thawed cell pellets using the innuPREP RNA Mini Kit (Analytik Jena AG, Jena, Germany), following the manufacturer’s instructions. Subsequently, the amount of RNA was determined by evaluating the absorbance at 260 and 280 nm with the Genesis 10 Bio photometer (Thermo Fisher Scientific, Waltham, Massachusetts, USA). The isolated RNA samples were used for generating cDNA. This was achieved with the SuperScript IV Reverse Transcriptase kit (Thermo Fisher Scientific, Waltham, Massachusetts, USA) by following the protocol of the manufacturer. If not immediately processed, cDNA samples were frozen at -20 °C for later usage. Previously generated cDNA samples were analyzed by reverse transcriptase (RT)-qPCR analysis. For this purpose, the cDNA was diluted to a final concentration of 50 ng/μL using nuclease-free water. This quantity was subsequently pipetted together with 3 μL 5 × SYBR green (Bio-Rad, Hercules, California, USA), 0.5 μL forward primer (10 μM) and 0.5 μL reverse primer (10 μM) on a 384-PCR well plate. The RT-qPCR was performed on a Thermocycler Quant Studio 5 (Thermo Fisher Scientific, Waltham, Massachusetts, USA) using a preset protocol with the corresponding 40 cycles PCR program: 95 °C—15 min, 95 °C—15 s, 60 °C—20 s, 72 °C -20 s, 95 °C—15 s, 60 °C—15 s and 95 °C—15 s. The subsequent data analysis was carried out on the QuantStudioTM Design & Analysis Software (Thermo Fisher Scientific, Waltham, Massachusetts, USA). GAPDH was used as an internal control. RT-PCR Primers used were as follows: *Cd24* forward ACATCTGTTGCACCGTTTCCCG, *Cd24* reverse CAGGAGACCAGCTGTGGACTG, *Gapdh* forward CTTCACCACCATGGAGAAGGC and *Gapdh* reverse GGCATGGACTGTGGTCATGAG.

### Melanin determination

Melanin content in the Susp and Adh population of B16-F10 cells were quantified as per the protocol described in [48]. For this purpose, 3D soft agarose culture assays were set up using phenol red-free RPMI (ThermoFischer Scientific, 11,835,030) complete cell culture medium. On the final day of the soft agarose colony formation assay, the culture supernatant was assessed for solubilized pigment levels at 490 nm using the ClarioStar system (BMG LABTECH GmbH, Ortenberg, Germany). The absorbance was averaged from three wells, and each experiment was performed in pentaplicates.

### DNA fragmentation assay

gDNA was extracted from cell pellets using the Wizard genomic DNA isolation kit (Promega Corporation, Fitchburg, Wisconsin, USA), according to the instructions of the supplier. The freshly isolated gDNA samples were quantified and stored at -20 °C for future experimental purposes. Agarose gel electrophoresis was used to separate previously isolated gDNA samples. To achieve this, a 1% agarose gel was prepared by mixing 1 g of agarose powder (Biozym Scientific GmbH, Oldendorf, Germany) with 100 ml of 1xTBE buffer. The mixture was heated for 1–2 min until the agarose was completely melted. Subsequently, 3 μL Gel Red Dye (Thermo Fisher Scientific, Waltham, Massachusetts, USA) was added to the solution. Thereafter, the agarose was poured into a gel tray and allowed to cool down. Following that, the gel was loaded with gDNA samples that had previously been mixed with 6xTriTrack DNA Loading Dye (Thermo Fisher Scientific, Waltham, Massachusetts, USA). The separation was conducted at 120 V for 1 h. Afterwards, gels were visualized by the INTAS gel IX Imager (INTAS Science Imaging, Göttingen, Germany).

### In vitro tumorigenicity assay

For soft agarose colony formation assays, Susp and Adh cells were suspended at a concentration of 1.5 million cells per mL in pH indicator Phenol-red dye-free RPMI media (ThermoFischer Scientific, 11,835,030) containing 0.33% low melting agarose (GoldBio. 1328 Ashby Road St Louis, A-204–25). 150,000 cells regardless of whether Trypan Blue positive or not were counted and mixed with pre-warmed 0.33% agar and plated on a bottom layer of media containing 0.6% agar in a 12-well plate. After an incubation at RT for 25 min, the soft agar culture plates were transferred to the cell culture incubator. The cells were cultured for 3 weeks before counting. Images of colonies were captured at room temperature using a camera equipped microscope with a 5 × /0.13 NA objective lens. No imaging medium was used, the culture plates were directly imaged. The images were cropped and contrast adjusted using Inkscape.

### Bromodeoxyuridine proliferation assay

A total of 10,000 Adh and Susp cells were seeded on a 96 well plate. The assay was carried out with the Cell proliferation ELISA, BrdU kit (Merck KgaA, Darmstadt, Germany), in accordance with the manufacturer’s protocol. Plates were analyzed by measuring the absorbance at 450 and 690 nm using the ClarioStar system (BMG LABTECH GmbH, Ortenberg, Germany).

### MTT assay

A total of 5000 Adh and Susp cells was seeded on a 96 well plate at least 16 h prior to adding the MTT reagent. The assay was performed with the CellTiter 96® Non-Radioactive Cell Proliferation kit (Promega Corporation, Wisconsin, USA) by following the protocol of the supplier. Afterwards, plates were analyzed by measuring the absorbance at 490 and 570 nm using the ClarioStar system (BMG LABTECH GmbH, Ortenberg, Germany).

### Statistical evaluation

The data presented here was statistically analyzed using GrahPad PRISM version 5.0 (Graphpad Software, San Diego, California, USA). An unpaired Student's t-test was the choice for analysis, with p-values less than 0.05 considered as significant. This was denoted by the corresponding asterisks within the figures (* *p* ≤ 0.05, ** *p* ≤ 0.01, *** *p* ≤ 0.001).

## Supplementary Information

Below is the link to the electronic supplementary material.

Supplementary file1 (PDF 1.37 MB)

## Data Availability

The data that supports the findings of this study are available in the supplementary materials of this article and/or from the corresponding author upon reasonable request. Additionally, specific flow cytometry datasets and raw western blot images will be made available in Figshare repository.

## References

[1] Damsky WE, Rosenbaum LE, Bosenberg M (2010) Decoding melanoma metastasis. Cancers (Basel) 3(1):126–163 24212610 10.3390/cancers3010126 PMC3756353

[2] Ugurel S, Gutzmer R (2023) Melanom. J Dtsch Dermatol Ges 21(4):343–347 36999586 10.1111/ddg.15053

[3] Fares J, Fares MY, Khachfe HH, Salhab HA, Fares Y (2020) Molecular principles of metastasis: a hallmark of cancer revisited. Signal Transduct Target Ther 5(1):28 32296047 10.1038/s41392-020-0134-x PMC7067809

[4] Valastyan S, Weinberg RA (2011) Tumor metastasis: molecular insights and evolving paradigms. Cell 147(2):275–292 22000009 10.1016/j.cell.2011.09.024 PMC3261217

[5] Ulaganathan VK, Ullrich A (2017) DNA-dependent protein synthesis exhibited by cancer shed particulates. BioRxiv (121186)

[6] Sun G, Guzman E, Balasanyan V, Conner CM, Wong K, Zhou HR et al (2017) A molecular signature for anastasis, recovery from the brink of apoptotic cell death. J Cell Biol 216(10):3355–3368 28768686 10.1083/jcb.201706134 PMC5626555

[7] Sun G, Montell DJ (2017) Q&A: Cellular near death experiences-what is anastasis? BMC Biol 15(1):92 29065871 10.1186/s12915-017-0441-z PMC5655817

[8] Berthenet K, Castillo Ferrer C, Fanfone D, Popgeorgiev N, Neves D, Bertolino P et al (2020) Failed apoptosis enhances melanoma cancer cell aggressiveness. Cell Rep 31(10):107731 32521256 10.1016/j.celrep.2020.107731

[9] Tang HL, Tang HM, Mak KH, Hu S, Wang SS, Wong KM et al (2012) Cell survival, DNA damage, and oncogenic transformation after a transient and reversible apoptotic response. Mol Biol Cell 23(12):2240–2252 22535522 10.1091/mbc.E11-11-0926 PMC3374744

[10] Tang HL, Yuen KL, Tang HM, Fung MC (2009) Reversibility of apoptosis in cancer cells. Br J Cancer 100(1):118–122 19088725 10.1038/sj.bjc.6604802 PMC2634673

[11] Jaggupilli A, Elkord E (2012) Significance of CD44 and CD24 as cancer stem cell markers: an enduring ambiguity. Clin Dev Immunol 30(2012):708036 10.1155/2012/708036 PMC3369436 22693526

[12] Tang M-R, Wang Y-X, Guo S, Han S-Y, Li H-H, Jin S-F (2014) CD24 expression predicts poor prognosis for patients with cutaneous malignant melanoma. Int J Clin Exp Med 7(11):4337–4341 25550951 PMC4276209

[13] Li W, Ma H, Zhang J, Zhu L, Wang C, Yang Y (2017) Unraveling the roles of CD44/CD24 and ALDH1 as cancer stem cell markers in tumorigenesis and metastasis. Sci Rep 7(1):13856 29062075 10.1038/s41598-017-14364-2 PMC5653849

[14] Hüser L, Sachindra S, Granados K, Federico A, Larribère L, Novak D et al (2018) SOX2-mediated upregulation of CD24 promotes adaptive resistance toward targeted therapy in melanoma. Int J Cancer 143(12):3131–3142 29905375 10.1002/ijc.31609

[15] Zhou M, Xie P, Chen L, Zhang P, Xu F (2023) Correlation between the expression of CD24 on circulating tumor cells and prognosis in breast cancer. Am J Transl Res 15(3):1941–1952 37056857 PMC10086875

[16] Knowles O, Doldan P, Hillier-Richardson I, Lunt S, Youssef G, Gammon L et al (2023) A CD24+CD271+ melanoma cancer stem cell generates a diffuse hierarchy of attributes that promote metastasis and therapeutic resistance. BioRxiv (2023.06.07.544036)

[17] Barkal AA, Brewer RE, Markovic M, Kowarsky M, Barkal SA, Zaro BW et al (2019) CD24 signalling through macrophage Siglec-10 is a target for cancer immunotherapy. Nature 572(7769):392–396 31367043 10.1038/s41586-019-1456-0 PMC6697206

[18] Panagiotou E, Syrigos NK, Charpidou A, Kotteas E, Vathiotis IA (2022) CD24: a novel target for cancer immunotherapy. J Pers Med 12(8):1235 10.3390/jpm12081235 PMC9409925 36013184

[19] Yang Y, Wu H, Yang Y, Kang Y, He R, Zhou B et al (2023) Dual blockade of CD47 and CD24 signaling using a novel bispecific antibody fusion protein enhances macrophage immunotherapy. Mol Ther Oncolytics 19(31):100747 10.1016/j.omto.2023.100747 PMC10689933 38046893

[20] Li X, Tian W, Jiang Z, Song Y, Leng X, Yu J (2024) Targeting CD24/Siglec-10 signal pathway for cancer immunotherapy: recent advances and future directions. Cancer Immunol Immunother 73(2):31 38279998 10.1007/s00262-023-03606-0 PMC10821995

[21] Huang S, Zhang X, Wei Y, Xiao Y (2024) Checkpoint CD24 function on tumor and immunotherapy. Front Immunol 29(15):1367959 10.3389/fimmu.2024.1367959 PMC10937401 38487533

[22] Hakami Z, Raposo T, Alsulaiman A, Dalleywater W, Otifi H, Horobin C et al (2021) *N* -glycosylation of CD24 mediates cell motility but inhibits cell proliferation in colorectal cancer. BioRxiv (2021.05.02.442315)

[23] Kinoshita T (2020) Biosynthesis and biology of mammalian GPI-anchored proteins. Open Biol 10(3):190290 32156170 10.1098/rsob.190290 PMC7125958

[24] Altevogt P, Sammar M, Hüser L, Kristiansen G (2021) Novel insights into the function of CD24: A driving force in cancer. Int J Cancer 148(3):546–559 32790899 10.1002/ijc.33249

[25] Low MG (1989) Glycosyl-phosphatidylinositol: a versatile anchor for cell surface proteins. FASEB J 3(5):1600–1608 2522071 10.1096/fasebj.3.5.2522071

[26] Taniuchi K, Nishimori I, Hollingsworth MA (2011) Intracellular CD24 inhibits cell invasion by posttranscriptional regulation of BART through interaction with G3BP. Cancer Res 71(3):895–905 21266361 10.1158/0008-5472.CAN-10-2743

[27] Bretz NP, Salnikov AV, Perne C, Keller S, Wang X, Mierke CT et al (2012) CD24 controls Src/STAT3 activity in human tumors. Cell Mol Life Sci 69(22):3863–3879 22760497 10.1007/s00018-012-1055-9 PMC11114558

[28] Baumann P, Cremers N, Kroese F, Orend G, Chiquet-Ehrismann R, Uede T et al (2005) CD24 expression causes the acquisition of multiple cellular properties associated with tumor growth and metastasis. Cancer Res 65(23):10783–10793 16322224 10.1158/0008-5472.CAN-05-0619

[29] Li D, Hu M, Liu Y, Ye P, Du P, Li C-S et al (2018) CD24-p53 axis suppresses diethylnitrosamine-induced hepatocellular carcinogenesis by sustaining intrahepatic macrophages. Cell Discov 6(4):6 10.1038/s41421-017-0007-9 PMC5799181 29423273

[30] Zhao W, Li Y, Zhang X (2017) Stemness-Related Markers in Cancer. Cancer Transl Med 3(3):87–95 29276782 10.4103/ctm.ctm_69_16 PMC5737740

[31] Al-Hajj M, Wicha MS, Benito-Hernandez A, Morrison SJ, Clarke MF (2003) Prospective identification of tumorigenic breast cancer cells. Proc Natl Acad Sci USA 100(7):3983–3988 12629218 10.1073/pnas.0530291100 PMC153034

[32] Xu H, Mu J, Xiao J, Wu X, Li M, Liu T et al (2016) CD24 negative lung cancer cells, possessing partial cancer stem cell properties, cannot be considered as cancer stem cells. Am J Cancer Res 6(1):51–60 27073722 PMC4759396

[33] Tang M-R, Guo J-Y, Wang D, Xu N (2018) Identification of CD24 as a marker for tumorigenesis of melanoma. Onco Targets Ther 12(11):3401–3406 10.2147/OTT.S157043 PMC6003289 29928131

[34] Wlodkowic D, Telford W, Skommer J, Darzynkiewicz Z (2011) Apoptosis and beyond: cytometry in studies of programmed cell death. Methods Cell Biol 103:55–98 21722800 10.1016/B978-0-12-385493-3.00004-8 PMC3263828

[35] Lakshmanan I, Batra SK (2013) Protocol for apoptosis assay by flow cytometry using annexin V staining method. Bio Protoc 3(6):e374 10.21769/bioprotoc.374 PMC4943750 27430005

[36] Galluzzi L, Vitale I, Aaronson SA, Abrams JM, Adam D, Agostinis P et al (2018) Molecular mechanisms of cell death: recommendations of the Nomenclature Committee on Cell Death 2018. Cell Death Differ 25(3):486–541 29362479 10.1038/s41418-017-0012-4 PMC5864239

[37] Li K, Li Y, Shelton JM, Richardson JA, Spencer E, Chen ZJ et al (2000) Cytochrome c deficiency causes embryonic lethality and attenuates stress-induced apoptosis. Cell 101(4):389–399 10830166 10.1016/s0092-8674(00)80849-1

[38] Anvekar RA, Asciolla JJ, Missert DJ, Chipuk JE (2011) Born to be alive: a role for the BCL-2 family in melanoma tumor cell survival, apoptosis, and treatment. Front Oncol 1(34):34 10.3389/fonc.2011.00034 PMC3260552 22268005

[39] Saraste A, Pulkki K (2000) Morphologic and biochemical hallmarks of apoptosis. Cardiovasc Res 45(3):528–537 10728374 10.1016/s0008-6363(99)00384-3

[40] Frisch SM, Francis H (1994) Disruption of epithelial cell-matrix interactions induces apoptosis. J Cell Biol 124(4):619–626 8106557 10.1083/jcb.124.4.619 PMC2119917

[41] Reddig PJ, Juliano RL (2005) Clinging to life: cell to matrix adhesion and cell survival. Cancer Metastasis Rev 24(3):425–439 16258730 10.1007/s10555-005-5134-3

[42] Rose JL, Reeves KC, Likhotvorik RI, Hoyt DG (2007) Base excision repair proteins are required for integrin-mediated suppression of bleomycin-induced DNA breakage in murine lung endothelial cells. J Pharmacol Exp Ther 321(1):318–326 17202402 10.1124/jpet.106.113498

[43] Dickreuter E, Eke I, Krause M, Borgmann K, van Vugt MA, Cordes N (2016) Targeting of β1 integrins impairs DNA repair for radiosensitization of head and neck cancer cells. Oncogene 35(11):1353–1362 26073085 10.1038/onc.2015.212

[44] Ogata M, Oomori T, Soga H, Ota Y, Itoh A, Matsutani T et al (2009) DNA repair after DNA fragmentation in mouse small intestinal epithelial cells. Cell Tissue Res 335(2):371–382 19015882 10.1007/s00441-008-0727-0

[45] Prates Mori M, de Souza-Pinto NC (2018) Role of mitochondrial dysfunction in the pathophysiology of DNA repair disorders. Cell Biol Int 42(6):643–650 29271530 10.1002/cbin.10917

[46] Tubio JMC, Estivill X (2011) Cancer: When catastrophe strikes a cell. Nature 470(7335):476–477 21350479 10.1038/470476a

[47] Ulaganathan VK, Vasileva MH (2023) A strategy for uncovering germline variants altering anti-tumor CD8 T cell response. J Genet Genomics 50(5):353–361 36690075 10.1016/j.jgg.2023.01.001

[48] Chung S, Lim GJ, Lee JY (2019) Quantitative analysis of melanin content in a three-dimensional melanoma cell culture. Sci Rep 9(1):780 30692593 10.1038/s41598-018-37055-y PMC6349835

