# Supplementary material for: CD24 flags anastasis in melanoma cells
Source: Apoptosis. 2024 Aug 13;30(1-2):1–15. doi: 10.1007/s10495-024-01990-1 (PMC11799124; doi:10.1007/s10495-024-01990-1)
Supplement: Supplementary file 1 — Supplementary file1 (PDF 1.37 MB) [file 10495_2024_1990_MOESM1_ESM.pdf]

Supplementary Information

CD24 flags anastasis in melanoma cells

Martina H. Vasileva[1], Anette Bennemann[1], Karolin Zachmann[1], Michael P. Schön[1,2], Jorge Frank[1,*], Vijay Kumar Ulaganathan[1,3,*]

1. Department of Dermatology, Venereology and Allergology, University Medical Center Göttingen, Göttingen, Germany
2. Lower Saxony Institute of Occupational Dermatology, University Medical Center Göttingen, Göttingen, Germany
3. University of Lorraine, NGERE Unit, Faculté de Médecine, 9 Avenue de La Forêt de Haye, Vandoeuvre-lès-Nancy 54505, France

* Equally contributing senior author;

*Pr. Dr. Vijay K. ULAGANATHAN, vijay-kumar.ulaganathan@uni-tuebingen.de

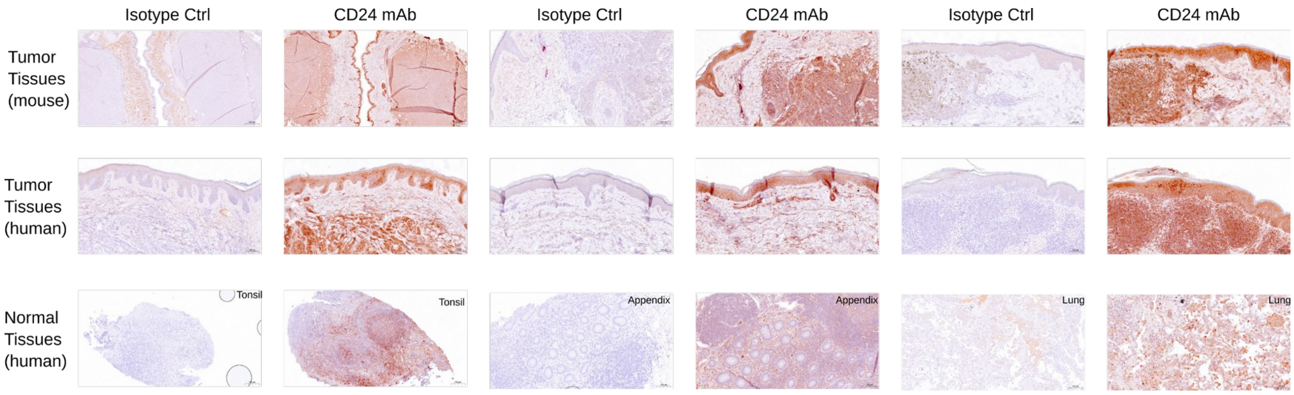

Supplementary Figure 1. Tumor and normal tissue expression analysis for CD24
Immunohistochemistry staining for CD24 in three independent tumor sections. Top panel: B16-F10 implanted tumors in C57BL/6 mice. Middle panel: Melanoma tumors from three patient tumor samples. Bottom panel: normal tissue sections from tonsil, appendix and lung. Scale bar (inset). 20X Magnification.

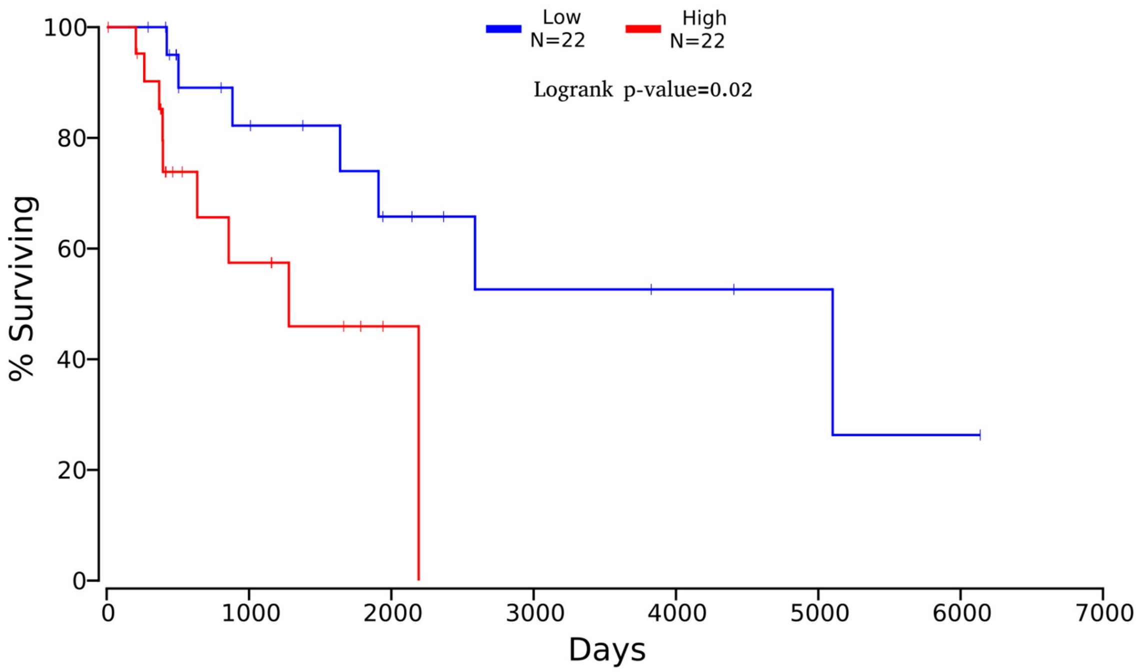

Supplementary Figure 2. Survival analysis of melanoma patients stratified by the levels of expression of CD24

The Kaplan-Meier survival plot shows the percentage survival versus time in days, with a log rank p-value for the analysis. The comparison is made between the subset of patients with very high expression levels of CD24 mRNA (>2400) and those with very low expression levels of CD24 mRNA (<1.75). Expression values are represented as the number of transcripts per million mapped reads. These subsets were selected from the top 5 percentile of the total SKCM cohort.

Data sourced from The Cancer Genome Atlas (TCGA) skin cancer (SKCM) cohort.

Raw Data Access: https://doi.org/10.6084/m9.figshare.25747077

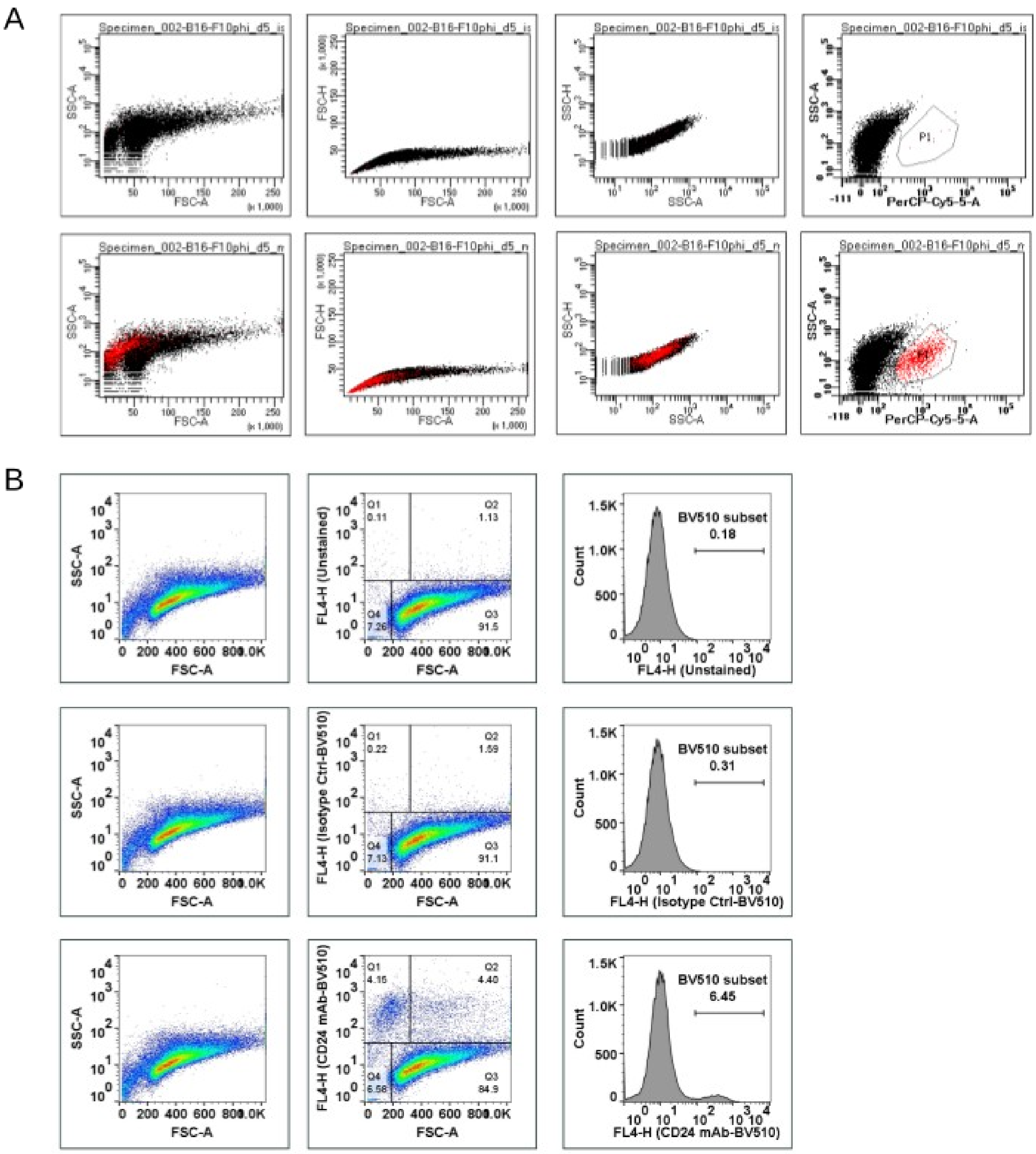

Supplementary Figure 3. Surface expression analysis of CD24 in a metastatic melanoma cell line. Flow cytometry dot plots illustrating the levels of CD24 surface expression on a distinct population of B16-F10 melanoma cells cultured in vitro. Data were obtained by flow cytometry surface staining of live B16-F10 cell confluent cultures using two different monoclonal antibodies recognizing mouse CD24. Panel A displays staining with clone M1/69, directly conjugated to PerCP-Cy5.5, while Panel B shows staining with clone 30-F1-BV510, directly conjugated to Brilliant Violet-510.

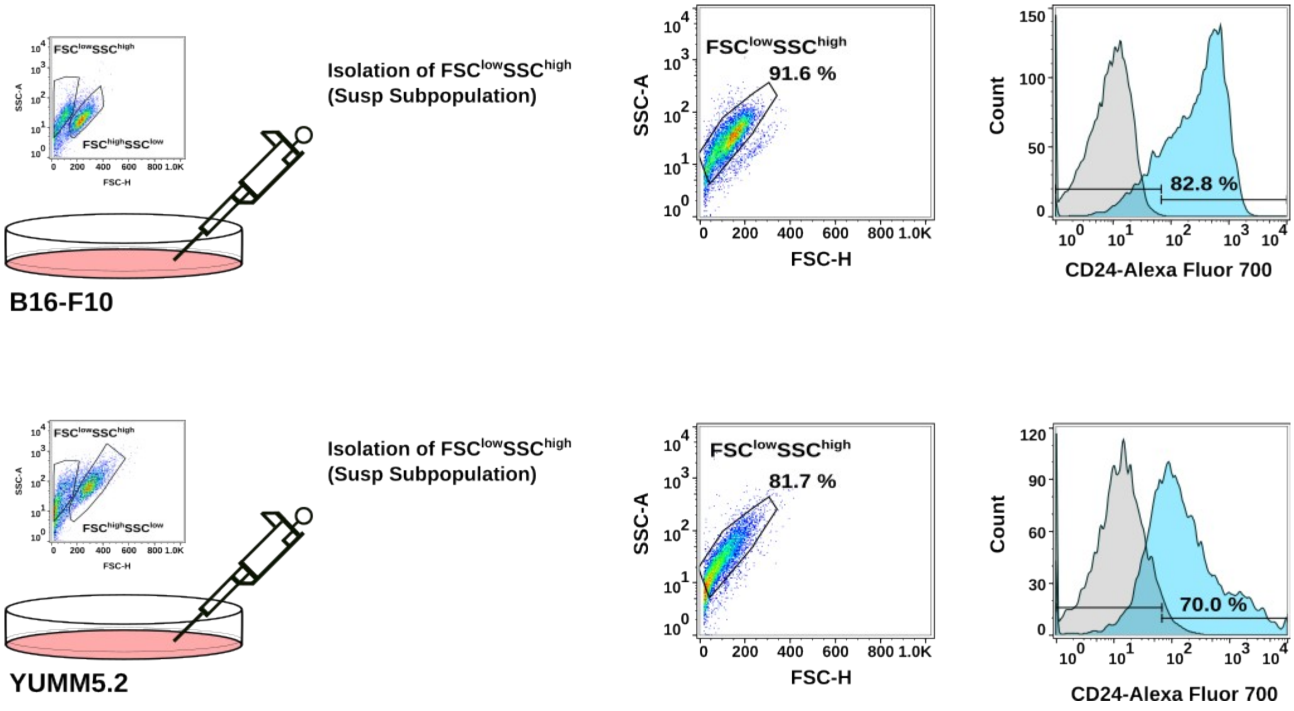

Supplementary Figure 4. Surface expression of CD24 in FSC^{low}SSC^{high} Susp Cells.
Non-adherent cells floating on top of B16-F10 (top panel) and YUMM5.2 (bottom panel) melanoma cell cultures were isolated and analyzed by flow cytometry after staining with either isotype control (grey histogram) or CD24 mAb Clone M1/69 (turquoise blue histogram).

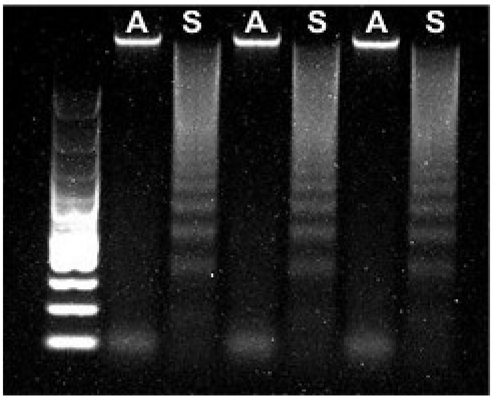

Supplementary Figure 5. Agarose gel electrophoresis of genomic DNA isolated from B16-F10 subpopulations
Agarose gel image, showing 3 independent experiments. A and S refer to Adh and Susp cells, respectively. Adh cells exhibit a distinct band pattern, typical for gDNA. Susp cells demonstrate DNA laddering as a result of cleavage, indicating cell death.

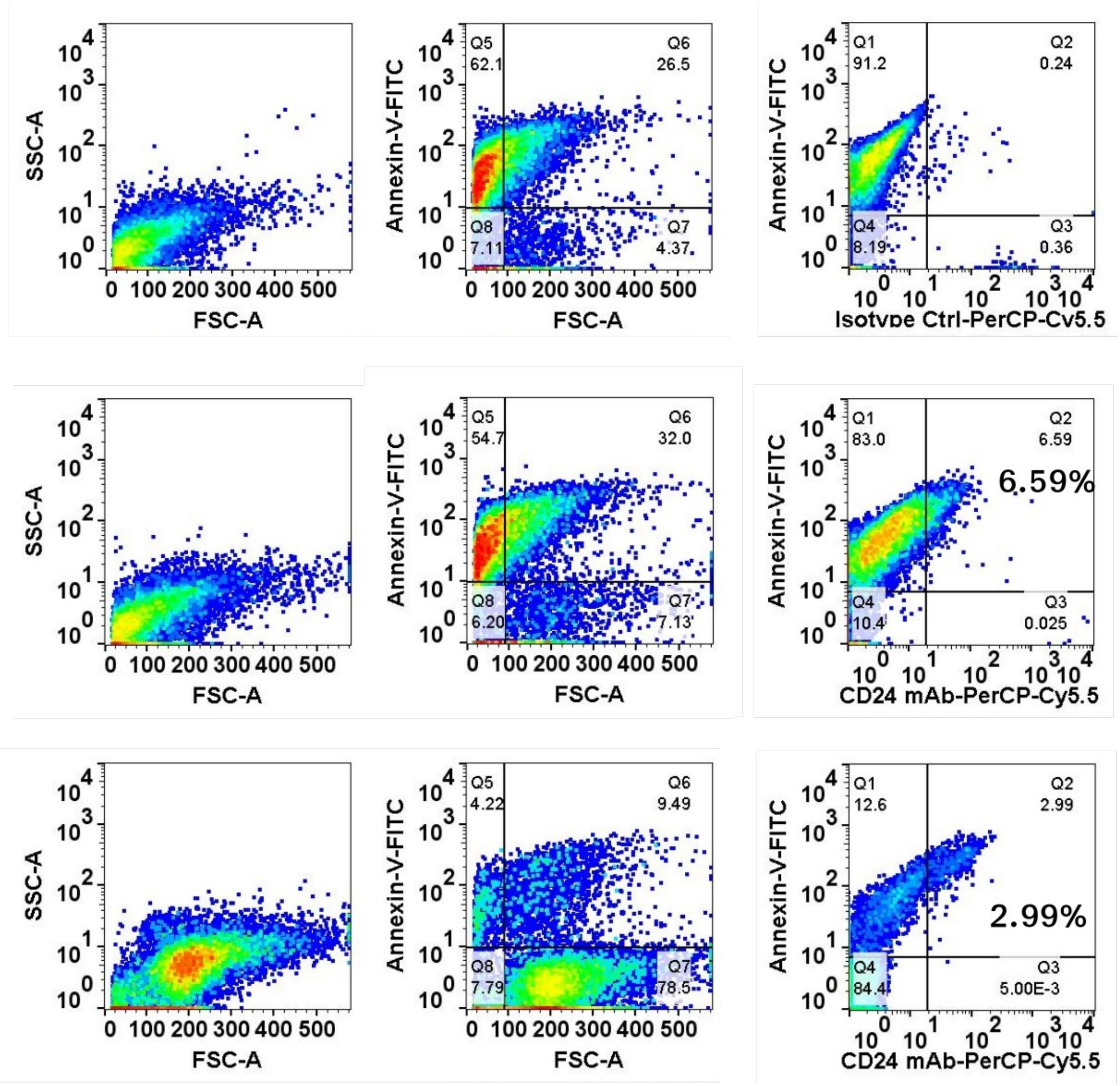

Supplementary Figure 6. Surface expression analysis of CD24 and apoptosis marker in B16-F10. Flow cytometry dot plots showing Annexin-V apoptosis marker staining and staining for CD24. The top row of the figure shows isotype control staining. The middle and bottom panels show staining for Annexin-V and CD24 in Susp and Adh subpopulations of cells from confluent B16-F10 culture, respectively.

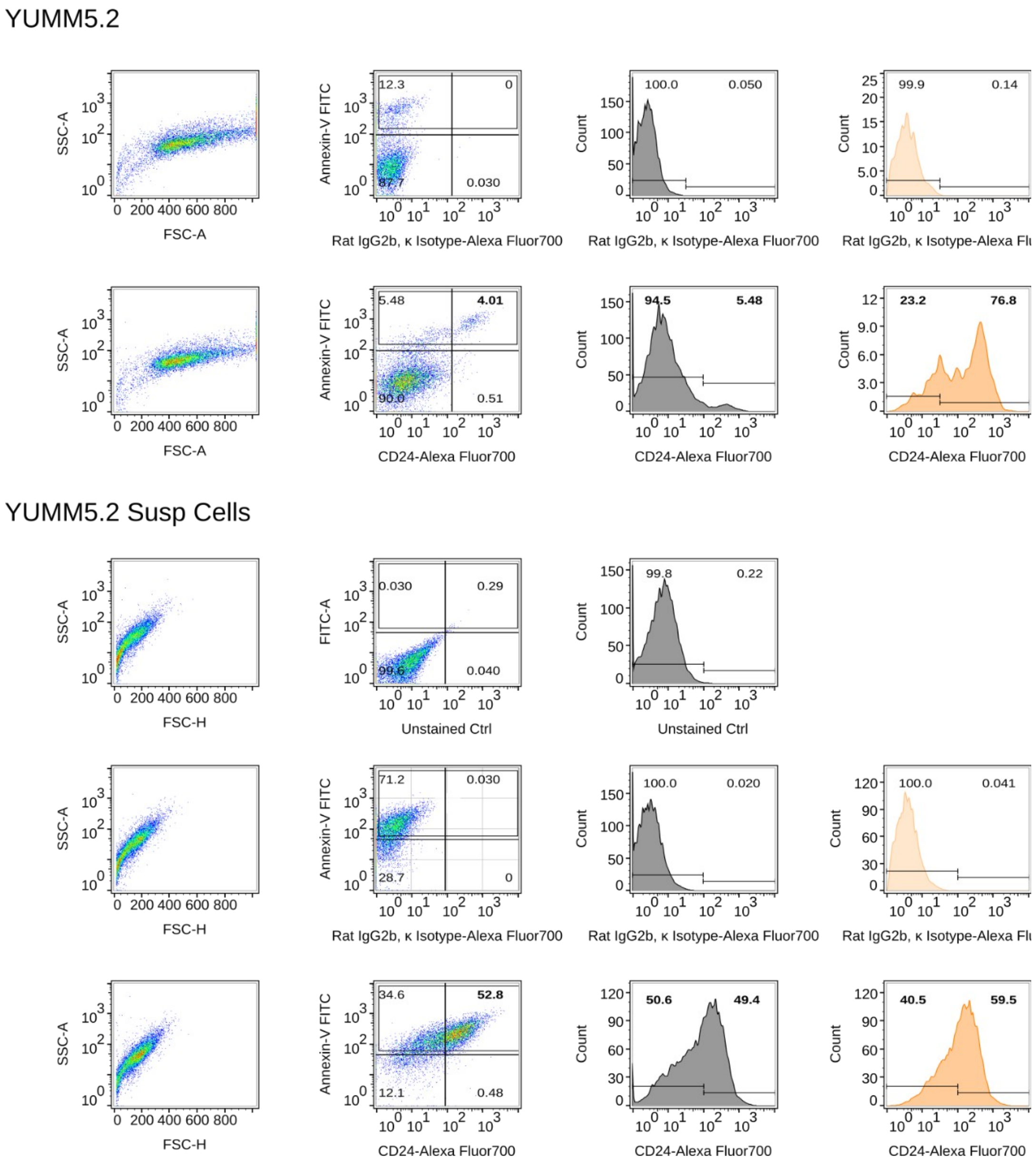

Supplementary Figure 7. Surface expression analysis of CD24 and apoptosis marker in YUMM5.2. Flow cytometry dot plots showing Annexin-V apoptosis marker staining and staining for CD24. The top row of the figure shows isotype control staining. The middle and bottom panels show staining for Annexin-V and CD24 in Susp and Adh subpopulations of cells from confluent YUMM5.2 culture, respectively.
